# The Influence of Visual Uncertainty on Word Surprisal and Processing Effort

**DOI:** 10.3389/fpsyg.2018.02387

**Published:** 2018-12-14

**Authors:** Christine S. Ankener, Mirjana Sekicki, Maria Staudte

**Affiliations:** Department of Language Science and Technology, Saarland University, Saarbrücken, Germany

**Keywords:** sentence processing, prediction, cognitive load, index of cognitive activity, entropy reduction, visual world paradigm, information theory, surprisal

## Abstract

A word’s predictability or surprisal, as determined by cloze probabilities or language models (Frank, 2013) is related to processing effort, in that less expected words take more effort to process (Hale, 2001; Lau et al., 2013). A word’s surprisal, however, may also be influenced by the non-linguistic context, such as visual cues: In the visual world paradigm (VWP), anticipatory eye movements suggest that listeners exploit the scene to predict what will be mentioned next (Altmann and Kamide, 1999). *How* visual context affects surprisal and processing effort, however, remains unclear. Here, we present a series of four studies providing evidence on how visually-determined probabilistic expectations for a spoken target word, as indicated by anticipatory eye movements, predict graded processing effort for that word, as assessed by a pupillometric measure (the *Index of Cognitive Activity*, ICA). These findings are a clear and robust demonstration that the non-linguistic context can immediately influence both lexical expectations, and surprisal-based processing effort.

## Introduction

The information-theoretic concepts of *entropy* (Shannon, 1949) and *surprisal* (Hale, 2001; Levy, 2008) have gained much attention in recent psycholinguistic research since they correlate with measures of processing effort (Demberg and Keller, 2008; Smith and Levy, 2013; Frank et al., 2015) and allow for quantitative predictions in language processing. One current approach is to derive surprisal from language models or cloze probabilities in order to quantify the amount of information conveyed by each word or linguistic unit. The surprisal values are then typically used as a predictor of processing effort experienced by the listener upon encountering these words or units (Demberg and Keller, 2008; DeLong et al., 2014). However, this approach inherently neglects the listener and the context at a particular point in time. This is especially important when considering a real-world situation, including a visual context in which language is used and processed. After all, seeing an object in the immediate surroundings can make the corresponding noun less surprising and more predictable than it would be given only the linguistic context. This paper hence considers a situated version of surprisal by combining linguistic surprisal with manipulated visual uncertainty about an upcoming referent in a co-present visual display. The corresponding effort is examined by additionally using a pupillary measure in the Visual World Paradigm (VWP) (Tanenhaus et al., 1995). Both the pupillary and the behavioral measures assessed effects of anticipation in the presence and absence of visual context. They reveal that listeners use the visual context to create probabilistic expectations for a target word and that this affects the processing effort induced by that target word accordingly. Results stress the importance of considering the relevant visual environment when using surprisal to quantify processing effort in situated language processing.

## Surprisal and Entropy Reduction in the VWP

Surprisal is often used to quantify the processing cost of linguistic units, as a reliable correlation has been established in that a more surprising and more informative word or utterance takes more time to read and/or more effort to process (Demberg and Keller, 2008; Smith and Levy, 2013). A similar connection has been found between effort and the concept of entropy reduction, according to which a more informative word reduces uncertainty about the entire sentence structure more sand hence might take longer to read (Linzen and Jaeger, 2014). This concept may also account for recent findings by Maess et al. (2016). In an MEG study, they found enhanced N400 activity for highly constraining verbs compared to unconstraining verbs as well as an inverse correlation between the verb constraint and the N400 on the subsequent noun.

As was the case in this study, it is currently common practice to compute linguistic surprisal from cloze studies, corpus data, or by means of language models. In situated communication, where a listener takes additional, non-linguistic cues such as the visual context into account, visual information influences the categorization of a piece of information as new and relevant. Here, visual cues also affect a word’s information and the associated processing cost, and, as a result, linguistic surprisal alone cannot account for a target word’s processing effort in such situations.

Adding visual context to an utterance further makes it easier to manipulate or establish a listener’s knowledge and expectations as she attempts to comprehend and interpret language. The VWP hence becomes an important tool when observing effects of simultaneously presented visual context. Of course, presenting listeners with visual objects that are congruent with what they hear causes them to consider what they see – i.e., as reflected by anticipatory eye movements as found by Altmann and Kamide (1999). Thus, the VWP always adds information and may therefore alter purely linguistic predictions.

We employed the VWP to investigate how different visual contexts influence expectations about upcoming nouns and the resulting effects on processing effort. Specifically, we examined whether listeners use verbal constraints to exclude visually given objects as upcoming referents when they do not match those constraints, thereby reducing referential uncertainty or entropy. In other words, while entropy reduction in purely linguistic contexts refers to a reduction of uncertainty about the rest of a sentence, in the VWP it refers to the reduction of visually presented target alternatives based on, for instance, a verb constraint. We further assessed whether this process of entropy reduction induces measurable effort on the verb and/or on the subsequent noun, which becomes more or less predictable as a result of this reduction. Thus, this work builds on the finding that anticipatory eye movements to particular referents indicate that their corresponding nouns are among the predicted target words – just like the “cake” during “eat” in Altmann and Kamide (1999) – and assumes that this can make them more or less surprising (see also Tourtouri et al. (2017) and Sekicki and Staudte (2018) for a related approach and similar measures).

### Index of Cognitive Activity

Anticipation in the VWP naturally requires listeners to move their eyes freely. With the *Index of Cognitive Activity* (ICA), we deployed a pupillometric measure which enables free eye movements while ensuring light insensitive online assessment of processing effort. That is, the measure has been shown to be robust with respect to eye movements and changes in luminance (Demberg and Sayeed, 2016). It is based on an analysis of pupil dilations, which have been reliably connected to cognitive activity due to language processing (Engelhardt et al., 2009). Pupils dilate either in reaction to task evoked mental effort (i.e., cognitive activity) or as a reflex, due to changes in light. When reacting to light, the eye’s circular muscles contract, while simultaneously the radial muscles prevent the pupil from dilating. In the case of reacting to mental effort, however, the radial muscles are activated, while the circular muscles are inhibited, both causing the pupil to dilate (Marshall, 2000). As a result, dilations due to mental effort are very short and abrupt movements of less than 0.5 mm in extent (Beatty, 1982; Lucero-Wagoner and Beatty, 2000). The ICA results from an analysis on pupil data that discards larger light-induced oscillations, while extracting the short and abrupt pupil jitter related to processing effort and are referred to as ICA “events” (see Marshall (2002) for a description of the method). The specific characteristics of those very short effort related dilations make the measure also less likely to be affected by artifacts due to gaze position in relation to the tracker lense. The filtered index then returns the exact times during the experiment at which such ICA events occur, with a resolution of 100 ms. For analysis, the number of events is then counted within a time period of interest.

Originally introduced as a measure of cognitive load in interactions with a visual display, the ICA has recently been proven to be reliable and responsive to cognitive effort induced by processing of language in different contexts. Demberg and Sayeed (2016), for instance, used the measurement in a series of reading and auditory experiments to show that the ICA can reliably reflect linguistic processing difficulty in different modalities. Additionally, their results reveal another important advantage, especially from a psycholinguistic point of view. They show that the ICA is robust with respect to eye movements, which makes it a handy measure of cognitive load to be employed in the VWP. This enables the experimenter to assess both visual attention and cognitive load simultaneously. We used the ICA in addition to traditional eye-movement analysis in order to measure cognitive load at the point of anticipatory eye movements, as well as on the target noun where expectations are verified and potential differences in surprisal can be expected.

Generally, high ICA values (i.e., more ICA events in a given time window) reflect higher cognitive effort, while low ICA values suggest comparatively less effort. In order to obtain ICA values, we used binocular eye-tracking at 250 *Hz* on an Eye-Link II tracker. The transformation and calculation of rapid small dilations from the tracker data was conducted in the *EyeWorks Workload Module* software (Version 3.12).

### Overview of the Paper

Before considering the ICA in the VWP to quantify the effect of the visual information on target word expectations, we set out to establish a baseline for processing effort of those target words in the absence of visual cues and as assessed by an already more established, behavioral reference measure (*Experiment 1*), as well as by the pupillary measure (*Experiment 2*).

Thus, the first two experiments were designed to assess surprisal-based processing effort in purely linguistic contexts in two different modalities and measures.

In *Experiment 1: Reading*, classical reading times of sentences with differently surprising verb-noun combinations (of the type: “The man spills/orders soon the water/ice cream/book”^1^) were considered. Differences were expected if predictability manipulations affected processing effort as assessed by this more traditional reference measure.

*Experiment 2: Listening* tested the same linguistic stimuli in auditory presentation mode, while measuring effort using the pupillary measure for the first time in the presented work. This study served as an intermediate step in assessing processing effort while changing the paradigm from *reading* to *listening*. The following two experiments employed the VWP to assess effects of added visual information on target word expectations, using the same sentences.

*Experiment 3: Listening and Viewing* observed whether surprisal of a word, as modulated by a combination of linguistic and visual context, predicted processing effort of that word.

Finally, *Experiment 4: Entropy Reduction* tested whether entropy reduction and/or surprisal of a word, as modulated *only* by visual context, predicted the critical word’s processing effort.

## Linguistic Materials: Design and Validation

### Design

The set of linguistic stimuli was held as similar as possible for all experiments (see Table 1 for an example) in order to keep results comparable. All items were German independent main clauses, uniform in their syntactic structure (NP-V-ADV-NP) and designed so that subjects did not contain any helpful information with respect to the expectancy of the verb or the noun.

**Table 1:** Sample items and corresponding pretest results for the two nouns in each verb condition: constraining (1) and unconstraining (2).

Verb Constraint	Noun	Plausibility *M (SD)*	Cloze % *M (SD)*
(1) The man *spills* soon the	(a) *water*	1.12 (0.68)	13.67 (18.06)^1^
	(b) *ice cream*	2.76 (2.17)	0.16 (0.54)
(2) The man *orders* soon the	(a) *water*	1.65 (1.50)	<0.01 (0.0)
	(b) *ice cream*	1.90 (1.80)	<0.01 (0.0)


*^*1*^Note that the relevant nouns were pre-selected from the DeReKo corpus (in order to control for frequency) and not collected from the cloze task answers. Hence the relatively high SD for these particular nouns in the cloze task.*

*Experiments 1* to *3* employed verbs from two categories, namely highly constraining^2^ (e.g., *spill*) or unconstraining (e.g., *order*). The different strengths of verb constraint made the verb arguments more or less predictable. *Experiment 4* used only highly constraining verbs in order to exclude any linguistic variation within an item.

The adverb following the verb (*The man spills soon the water*) served as a padding region, giving the listener more time to generate expectations about the upcoming object noun.

Both verb categories were additionally paired with two different object nouns [see Table 1, (1) and (2)], one of which is more plausible in the highly constraining verb context (see Table 1, column 3).

All stimuli were recorded using *Audacity* (Version 2.0.6). Factors with potentially substantial influence on processing effort, such as word length and frequency (Schilling et al., 1998), were controlled for. In particular, noun frequencies were derived *a priori* from word lists DeReWo^3^ of the German research corpus (DeReKo) and held approximately constant within an item. Differences in word length were integrated in the analyses (by either including the length factor as predictor in the model, or using mid-word time windows; see the *“Analysis”* subsections for details).

Fillers were always plausible sentences with differing length and of differing syntactic structure in order to prevent fatigue effects. Half of the fillers were followed by yes/no comprehension questions (such as “Did the man spill the lemonade?”) to keep participants focused.

### Validation

Both manipulations, i.e., strength of verb constraint and the nouns’ plausibility in their contexts, were validated offline prior to using the stimuli in the actual online experiments.

#### Verb Constraint

The more constraining a verb is, the fewer plausible continuations it allows. Hence, a classical sentence completion task for cloze probability assessed to what extent the verbal constraint increased the predictability of the target noun (Table 1, column 4)^4^. Seventeen German native speakers participated voluntarily in this online questionnaire. All items were truncated prior to the target noun and presented in one list, containing 50% fillers, shown in randomized order. Participants were asked to spontaneously complete the sentences with the noun best fitting the sentence context. Unique participation of each webform user was controlled for. Results from the cloze task showed differences between nouns in the highly constraining verb context only [see Table 1, column 4, (1a vs.1b)]. Cloze probabilities ranged from 4 to 55% for plausible nouns in highly constraining verb contexts (*spill water*) and from 0 and 4% for the less plausible nouns in the same contexts (*spill ice cream*). Unconstraining verbs produced cloze probabilities < 0.01 for all critical nouns. In sum, higher constraint led to higher cloze probabilities of the two subsequent nouns (see Table 1, column 4).

#### Verb – Noun Plausibility

A plausibility rating on a seven-point Likert scale assessed how plausible participants would rate a target noun to be in its sentence context (Table 1, column 3). 14 German native speakers participated voluntarily in the online questionnaire. Participants read the stimuli sentences in a webform and were asked to spontaneously judge the plausibility of each sentence combination, resulting from the Verb–Object manipulation, ranging from 1 (very plausible) to 7 (not plausible at all). Items were presented in randomized lists, containing 50% filler sentences. Each participant had only a single access to the webform. Results showed that plausibility – or thematic fit – of the nouns differed in the context of high constraining verbs [see Table 1, column 3, (1a vs.1b)], while both nouns were equally plausible in the unconstraining verb context [see Table 1, column 3, (2a vs.2b].

## Experiment 1: Reading

### Methods

The first experiment assessed effects of *purely linguistic* context on processing effort of the critical words, in terms of reading times, hereby employing a more traditional measure before using the ICA. An “implausible” condition as well as a spill-over region following the verb’s argument in all conditions were added only in this experiment (e.g., *the man soon spills the book at the restaurant*). The implausible condition served as a sanity check for design as well as for measure and was expected to elicit longer reading times for the highly surprising noun (*the book*). The added adverbial phrases served as post target spill-over regions for the time-dependent measure, which requires longer time windows (as opposed to ERPs or the ICA). The manipulation resulted in a 2 × 3 design in which constraining (*spill*) and unconstraining (*order*) verbs were paired with objects that were more plausible in the constraining verb context (*water*), less plausible (*ice cream*) and implausible (*book*). In the unconstraining verb context, all three objects were equally plausible, while the target noun’s plausibility differed in the constraining verb context.

Thirty-six experimental and thirty-six filler items were distributed across six lists, using the Latin square design in such a way that each participant would see each item in only one condition. Twenty-four native speakers of German (students of Saarland University) gave informed consent before participating in this study for monetary reimbursement. Their age ranged from 18 to 32 years (*M* = 22.71).

Sentences were presented as a whole, in the center of the screen (Times New Roman, 20 pt), with a drift correct fixation point, shown at the top left corner in order to avoid initial fixations at the sentence. Participants were instructed to read for comprehension, at their own pace.

### Predictions

Along with, for instance, Ferreira and Clifton (1986), who suggested that (in this case, syntactic) predictions affect reading times, or Rayner and Well (1996), Van Berkum et al. (2005), as well as Smith and Levy (2013), who propose that expectation-inconsistent words cause longer reading times, we expected predictability effects to be reflected in our behavioral measure. That is, higher (surprisal-based) processing effort, i.e., longer reading times, were expected on or after the implausible target nouns, only when following the constraining verbs. If, however, the verbal constraint alone was not enough to elicit (lexical) expectations about the target nouns, no differences between the object conditions in the constraining verb context were expected.

### Analysis

Statistical analyses of the data, collected in this and the following studies, were conducted using the R statistical programming environment (R Core Team, 2013) and the *lme4* package (Walker et al., 2015). Reading times were measured and analyzed on the verbs and target nouns as the critical regions, as well as on the respective spill-over regions. Time measures were log-transformed and entered as dependent variables into linear mixed-effects models. The contrast-coded Object and Verb conditions as well as the scaled length of the target word (measured in characters) were entered as fixed factors. Item and Participant ID were included as random effects. The models were run with the maximal converging random effects structure, including intercepts and slopes for both, Subject and Participant ID. The full models are available in the Supplementary Material.

### Results

Total dwell-time (in milliseconds and then log-transformed) on the critical region (the noun, as defined by a default AOI spanning the entire word) showed a significant difference only for the implausible condition, in orthogonal comparison to the less plausible condition (*spill the ice cream* (*M* = 6.09, *SD* = 0.55) vs. *spill the book* (*M* = 6.25, *SD* = 0.62, *p* < 0.005), only if the verb was constraining (*spill*; significant interaction with Verb, *p* < 0.05). No difference was found between the *more* and the *less plausible* conditions (*spill the water* vs. *spill the ice cream*). No significant results were found for the verb. Further, analysis of the first-pass measurement did not yield significant results. Regressions to the pre-target region showed the same pattern as total dwell-time. Analyses of the spill-over region showed no significant effects.

### Discussion

Results show that, in the absence of visual context, verbs with different strengths of constraint did not differ in processing effort. Moreover, the noun’s processing effort was only significantly affected when the respective noun was implausible in its linguistic context and hence unexpected, i.e., highly surprising.

This may appear surprising, given that several previous studies found effects of predictability and plausibility in reading or listening, as, for instance, Kutas and Federmeier (1999), or also Rommers et al. (2013), who found a graded N400 effect in response to more or less expected target words, as well as Rayner et al. (2004), who find an immediate effect of implausible words on eye movements in a reading task, but only delayed effects of smaller magnitude for less severe violations of plausibility. While one explanation for the null results could be a lack of power, it is important to note that we replicated these results in not only the subsequent experiments, but also in an additional self-paced reading study which is not reported in this paper. Thus, we propose that the null result is true and that it reflects the low differences in cloze probabilities between conditions (compared to, for instance, Rommers et al. (2013), where words with either very high or zero cloze probability were used). Further, the stimuli sentences were not embedded in wider contexts; hence no additional information, apart from the comparatively low verb constraint, was given. That is, no further information was available for the listener to form (lexical) expectations about the target noun. We suggest that in our case, verb constraints alone did not elicit concrete lexical predictions about the target nouns (beyond a semantic category). Compared to the verbs used in Maess et al. (2016), verbs in our study were less constraining, which could explain the lack of an effect on the critical word’s and spill-over region’s reading times. Whether these findings would be replicated within a different presentation mode and – not necessarily time-dependent^5^ – measure was tested in *Experiment 2*.

## Experiment 2: Listening

### Methods

The second experiment presented stimuli that were held as similar as possible to the ones used previously (without the implausible condition and the spill-over regions) in a different modality, namely auditorily, while assessing processing effort using a pupillary measure. Here, we (a) established a baseline for processing effort of the critical words in the absence of visual context in the respective measure, and (b) tested whether the results from the previous experiment were attributable to either the presentation mode (written) or the sensitivity of the time-dependent measurement (reading times), or indeed to the low overall predictability for the critical nouns.

All 36 native speakers of German, who participated in this study, gave informed consent and had not participated in the previous study. They were, again, monetarily reimbursed for their contribution. All were students of Saarland University and their age ranged from 19 to 46 years (*M* = 24.72). 20 experimental items in four conditions [*highly constraining* or *unconstraining* verbs, crossed with *more plausible or less plausible* objects (see Table 1)], as well as 26 filler items were used in each of the four lists for this experiment. Even though no visual stimulus was presented, participants’ eyes were tracked while they looked at a blank screen in order to extract the ICA values from the pupil jitter.

### Predictions

We previously suggested that our verbal constraints alone did not contain enough information to cause listeners to have lexical expectations about target nouns, resulting in the same processing effort for more or less constraining verbs and more or less plausible object nouns following those verbs. If this result was, however, due to the presentation mode (written) or the sensitivity of the time-dependent measurement (reading times), we expected to find differences in processing effort, as assessed by the ICA, for the same stimuli when presented auditorily. In that case, a lack of effects between the verbs and the plausible and implausible nouns following the constraining verbs in *Experiment 1* could not be due to the nature of expectations in the purely linguistic context and surprisal-based effort itself.

### Results

Demberg and Sayeed (2016) analyzed ICA events within a time window taken 600–1200 ms from the onset of the critical word. To compensate for the differences in target word length, we analyzed ICA event counts within a 600 ms time window, starting from the middle of the critical word’s duration (as in Sekicki and Staudte, 2018). This resulted in identical 600 ms time windows starting at a point where participants are considered to have identified the respective word. Word length was therefore not included as a covariate in the statistical models used to analyze the data from the ICA studies^6^.

**FIGURE 1:**
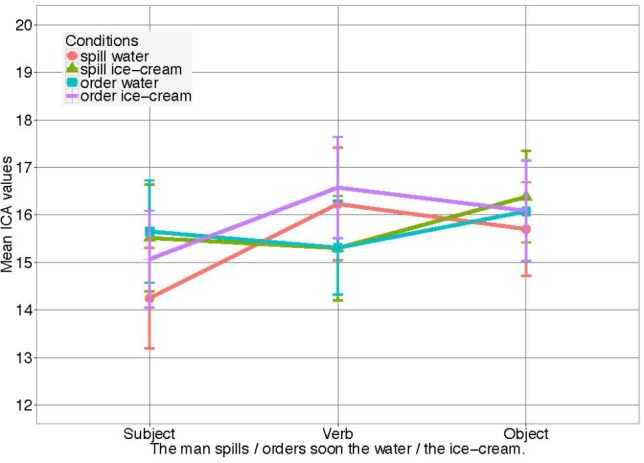
ICA Results for *Experiment 2* in all four conditions. Error bars reflect 95% confidence intervals (CI). For the models see Supplementary Materials.

Index of Cognitive Activity event counts were obtained for both eyes separately, but were summed for analysis since we are not aware of any theoretical reason why differences should be expected for the two eyes (Demberg and Sayeed, 2016). Data from both eyes (per 100 ms) were then summed for the entire 600 ms time windows. ICA events during the two critical time windows (verb and noun) were the basic dependent variable. Since those events were treated as a count variable, generalized mixed effects models with Poisson distribution were used. All independent variables were contrast coded for the analysis. Again, the verb and object manipulations (i.e., *order water/ice cream* and *spill water/ice cream*) did not result in differences in effort on the noun, as assessed by the ICA. Figure 1 shows how both verb conditions cause almost identical average cognitive load on the noun (*spill water/ice cream, M* = 16.05, *SD* = 6.51 vs. *order water/ice cream, M* = 16.07, *SD* = 7.09). That is, although measure (ICA) and modality (auditory) were different, as compared to the previous experiment, again no effect of the relatively mild verbal constraints on prediction and processing effort was observed in any of the conditions in contexts where no visual context information was presented.

### Discussion

Reading time results from *Experiment 1* were replicated in the pupillary measure. We hence suggest that the lack of differences between conditions was due neither to the measure, nor to the presentation mode. Rather, it can be explained by the verb not containing enough information for the listener to concretely expect the target noun on the lexical level. Results from *Experiment 2* further mark the baseline for processing effort in the absence of any visual context. The baseline shows how both verb and noun conditions require equal processing effort in a purely linguistic context. This observation is interpreted to be in accordance with Wlotko and Federmeier (2015), in the sense that they propose that the brain might not always engage in prediction to the same degree in all contexts and circumstances. The authors suggest that, instead, predictions (as in purely linguistic contexts) could be flexibly implemented in adaption to factors such as timing, availability of processing resources, or the informativity of contextual cues. As mentioned previously, in the case of the presented study, this then means that the weakly constraining linguistic contexts did not provide enough information for participants to make predictions beyond rough semantic categories.

The following two experiments introduce additional information in form of simultaneously presented visual context in order to observe how it influences expectations and the target word’s processing effort.

## Experiment 3: Listening and Viewing

### Visual Materials: Design and Validation

#### Design

**FIGURE 2:**
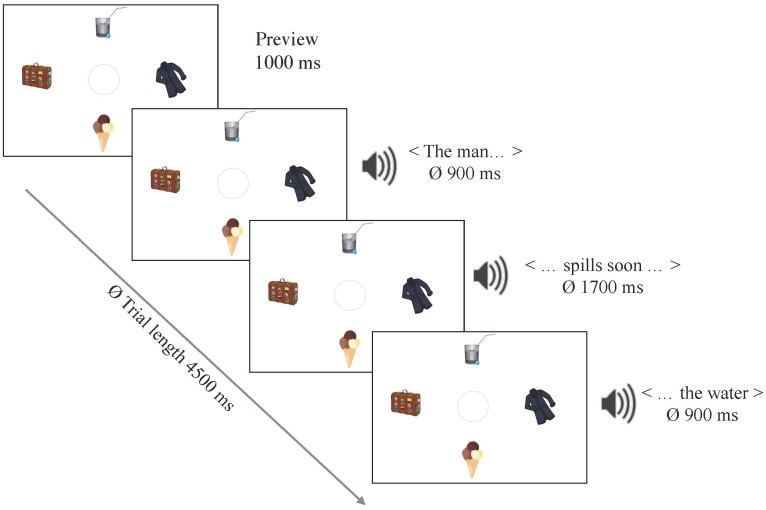
A trial time line and stimulus example for *Experiment 3.*

All scenes presented in the following two VWP studies consisted of four simple pieces of clip art, arranged around the screen center. One of the four objects corresponded to the target mentioned in the sentence. A second object was a competitor, matching the verb constraint only to some extent (less plausible), while the remainder were non-matching distractors (see Figure 2 for a sample display). All four objects matched the category introduced by the unconstraining verb. None of the objects in a display corresponded to the sentences’ highest-cloze nouns. Clip art items within one visual display were of similar complexity, uniformly salient in terms of colors, and depicted concrete and inanimate objects. The scenes were counterbalanced between two items. Positions of targets, competitors and distractors were rotated. Filler trials introduced variation in terms of the number of categories displayed (i.e., edible, drinkable, or wearable objects, but also drivable or ironable ones etc.).

#### Validation

All clip art used in the following studies was pre-tested for naming to make sure all of the objects were recognizable and well distinguishable. A control of the scenes for differences in luminance was not necessary due to the ICA’s robustness with respect to changes in light. A naming test was performed online, where the unique participation of each user was controlled for. All clip art was presented in two randomized lists. Participants were asked to spontaneously write down the name of the object they saw in the picture. Twenty-four people participated in this naming task. Pictures were used in the experimental items only if they were recognized reliably (>90% of participants recognized each object correctly).

### Methods

By adding visual context to the same stimuli used in the previous experiments, we now assessed the immediate effects of visual context on predictability and processing effort, as predicted by surprisal, for a spoken target word. We further observed whether anticipatory eye movements are relatable to differences in actual processing effort of the verb, e.g., due to reduction of visual entropy.

The linguistic stimulus set, manipulation and design were identical to *Experiment 2*. The simultaneously presented visual stimuli were arranged as shown in Figure 2 and functioned as an enhancement of both manipulations on the linguistic level (plausibility and verb constraint) by decreasing the number of potential target object options from a non-assessable number of nouns matching the verb in *Experiment 2*, to a countable number of options in the display.

Visual displays were presented from 1000 ms before sentence onset and during the whole sentence. Participants were asked to interact naturally with the scenes, not forcing themselves to look at or away from items. Their eyes were tracked in order to obtain eye movement data and extract the ICA values from the same data set. Thirty-six native speakers of German (all students of Saarland University) between 19 and 38 years of age (*M* = 23.25), all of whom had not participated in any of the previous lab experiments, were tested and received payment for participation. Data from two participants had to be excluded from the analysis due to technical problems.

### Predictions

We expected to replicate verb-driven anticipatory eye movements toward depicted target options – typically found in such setups, e.g., by Altmann and Kamide (1999), or Kamide et al. (2003) – as listeners exploit visual context information to expect the target word. If visual context information significantly influenced predictability and surprisal, and hence processing effort of the target nouns, we further expected lower ICA values in the case of the more plausible noun following the constraining verb. In other words, the more predictable and hence less surprising a noun was in its multimodal context, the easier it should be to process it, eliciting lower ICA values.

If anticipatory eye movements at the verb are relatable to differences in processing effort – possibly due to the listener excluding distractors from the set of possible target options – we further expected differences in the ICA on this region, namely higher values, as more options can be excluded. This would indicate that higher reduction of visual uncertainty, or entropy, would require more processing effort. Additionally, when comparing results from this study to those gathered in the previous experiment (i.e., results from purely linguistic vs. results from multi-modal contexts) an overall higher processing effort (i.e., higher ICA values) throughout the entire trial was expected if processing effort increases with the amount of information presented *simultaneously*. That is, as opposed to linguistic information which is assessed *sequentially*, one unit at a time, visual information allows for a *sudden* assessment of information which then has to be processed simultaneously with the linguistic information. This could cause overall processing effort to be higher as two modalities have to be processed and evaluated, compared to the processing and evaluation of just one (sequential) modality.

### Results

#### Eye Movement Data

**FIGURE 3:**
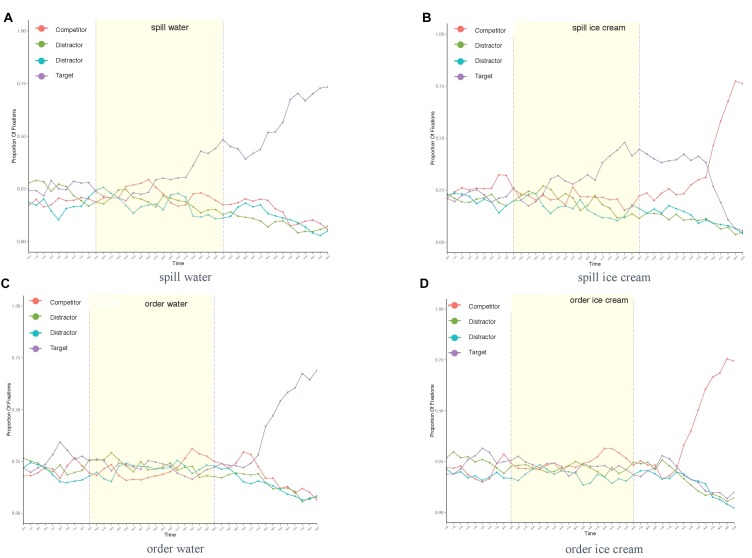
Proportion of fixations across trial length in all conditions *of Experiment 3*: spill water **(A)**, spill ice cream **(B)**, order water **(C)**, and order ice cream **(D)**.

For presentation purposes, fixation plots of the following studies show the overall fixation distribution across an example trial of averaged length in all conditions. Dashed lines mark the area of interest for eye movements to potential target options in anticipation of the noun, that is, the verb onset on the left and the noun onset on the right. Figure 3 shows increased anticipatory eye movements toward the object best matching the verb in the constraining verb condition (*spill*). At the same time, no such preference for any of the displayed objects was found in the unconstraining verb condition (*order water/ice cream*: see Figures 3C,D), suggesting that based on the context information, none of the objects could be specifically expected.

Statistical significance was assessed by analyzing differences in new inspections (i.e., the first in a series of inspections toward a region during the time periods of interest) between conditions. That is, we compared probabilities of verb-driven attention shifts toward objects matching the verb constraint.

In the verb window, looking toward the target object region (*water*) was significantly less probable if the verb was unconstraining compared to when the verb was (highly) constraining: *order* (*M* = 0.11, *SD* = 0.32) vs. *spill* (*M* = 0.16, *SD* = 0.37), β = -0.378, *SE* = 0.093, *z* = -4.038, *p* < .001. Further, inspections toward objects not corresponding to the target noun were significantly more likely if the verb was unconstraining: *order* (*M* = 0.34, *SD* = 0.47) vs. *spill* (*M* = 0.3, *SD* = 0.46), β = 0.141, *SE* = 0.071, *z* = 1.985, *p* < .05. In the noun region, the data shows that participants were significantly more likely to direct a new inspection to the more plausible object than to any other object in the scene.

#### Index of Cognitive Activity

**FIGURE 4:**
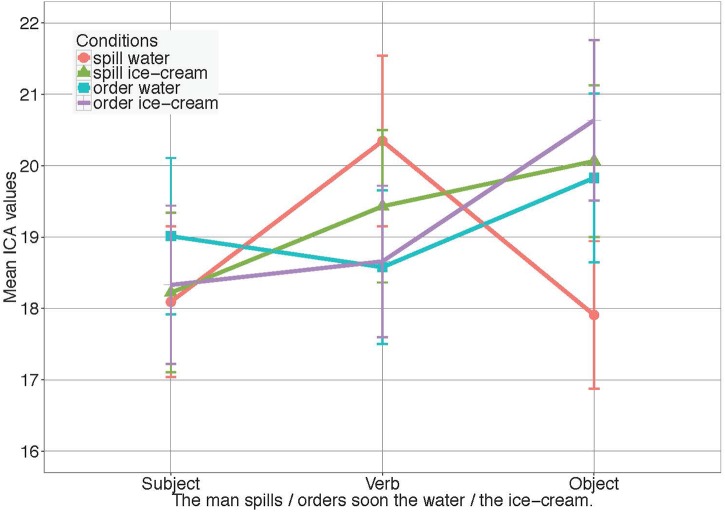
ICA Results for *Experiment 3* in all conditions. Error bars reflect 95% confidence intervals (CI). For the models see Supplementary Materials.

To assess effects of visual context on expectations and surprisal-based processing effort, again ICA event counts were analyzed within a 600 ms time window, starting from the middle of the critical word’s duration. ICA events obtained within the two critical time windows (see Figure 4) were used as the basic dependent variable in generalized mixed effects models (see 3 for the full model). Subjects and items were included as completely crossed random factors.

No significant effects were found in the verb window, where eye-movement data showed clear effects of anticipation. Only a non-significant trend toward higher ICA values in the case of higher entropy reduction, that is, for the highly constraining verb, was observed.

Values from the noun window, however, showed a significant interaction of *Verb* and *Object* (β = -0.071, *SE* = 0.035, *z* = -2.05, *p* < 0.05), as well as a significant main effect of *Verb* (*spill M* = 18.99, *SD* = 6.99 vs. *order M* = 20.24, *SD* = 7.7, β = 0.063, *SE* = 0.032, z = 1.98, *p* < 0.05). This suggests that *water* and *ice cream* affect processing effort to a different degree when succeeding the constraining verb *spill* than they do when following the unconstraining verb *order*.

Planned pairwise comparisons revealed a significant effect of *Noun* in the case of the constraining verb *spill* (*water M* = 17.91, *SD* = 6.91, *ice cream M* = 20.06, *SD* = 7.07, β = 0.113, *SE* = 0.045, *z* = 2.51, *p* < .05), but not for the unconstraining verb *order* (*water M* = 19.83, *SD* = 7.91, *ice cream M* = 20.64, *SD* = 7.49, *p* = 0.46), implying that *water* was easier to process than *ice cream* only when following *spill*. In line with this, a significant effect of *Verb* was found, in the case of the slightly preferred object *water* (β = 0.094, *SE* = 0.039, *z* = 2.41, *p* < 0.05), but not for *ice cream* (*p* = 0.675).

#### Experiment Comparison

Index of cognitive activity values collected within the critical time windows in *Experiments 2* and *3* were compared as a merged dataset in order to assess whether processing effort rises as more information is presented simultaneously (i.e., to compare results from two ICA studies with and without visual context). Each experiment’s ID (*2* vs. *3*), as well as Verb and Object conditions were entered into the generalized mixed effects models as contrast coded fixed factors.

**FIGURE 5:**
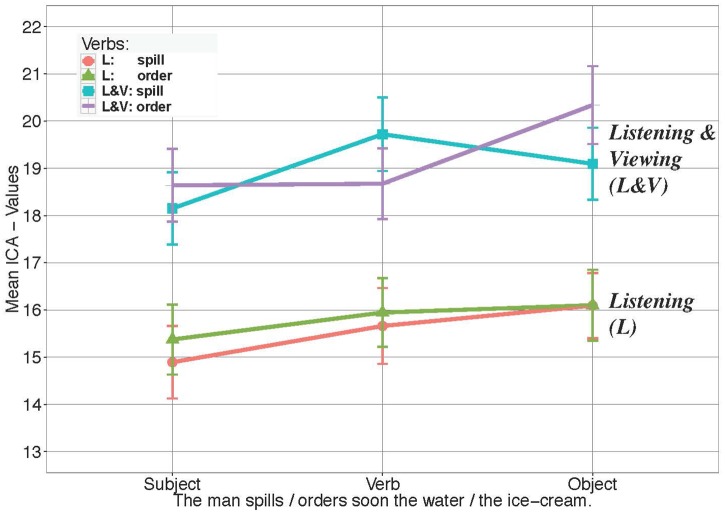
Comparison of ICA values in *Experiment 2 and Experiment 3.* Both object nouns of a verb condition are considered together for the plot. Graphs show the significant main effect of *Experiment* and a significant interaction of *Verb* and *Experiment.* Error bars reflect 95% confidence intervals (CI). For the models see Supplementary Materials.

Figure 5 shows overall higher processing effort if additional visual context had to be processed along with the utterance.

In accordance with the graph, the models fitted for the ICA events revealed a highly significant main effect of *Experiment* in the verb window (*Experiment 1: M* = 15.80, *SD* = 7.25 vs. *Experiment 2: M* = 19.19, *SD* = 7.25, β = 0.22, *SE* = 0.020, *z* = 10.7, *p* < 0.001) and noun window (*Experiment 1: M* = 16.06, *SD* = 6.79 vs. *Experiment 2: M* = 19.76, *SD* = 7.41, β = 0.262, *SE* = 0.020, z = 13.05, *p* < 0.001), showing that overall processing effort was significantly higher when visual context was presented.

We further found a significant interaction of *Verb (constraint)* and *Experiment* in the verb (β = -0.008, *SE* = 0.029, *z* = -2.82, *p* < 0.005) window. The same interaction was also found in the noun window (β = 0.066, *SE* = 0.032, *z* = 2.05, *p* < 0.05). In both cases, follow up comparisons revealed however, that the interaction was carried by the opposite direction of the non-significant trend between the two verbs in the two studies. That is, compared to the unconstraining verb (*order*), the constraining verb (*spill*) showed the tendency to require more effort to process in *Experiment 3*, where visual context was given, while requiring slightly less effort in *Experiment 2*, in the absence of visual context.

These results imply that target nouns were only more predictable in the context of the constraining verb, as compared to the unconstraining one, if visual context information was available. They further suggest that processing effort indeed increases exponentially with the amount of information presented simultaneously.

### Discussion

As expected, eye movement data revealed listeners’ use of verbal constraints mapped to visual context to expect the noun. That is, in replication of Altmann and Kamide (1999), our data showed that participants were more likely to fixate objects matching the verb in the time window prior to the noun.

The pupillary measure simultaneously revealed differences in surprisal-based processing effort on the noun as a result of the additional visual context. We found that processing effort differed between both noun conditions (*water* vs. *ice cream*) in cases when the verb was highly constraining (*spill*). No such difference was found after unconstraining verbs (*order*) carrying less information to expect the noun from the visual context. Note that the same linguistic stimuli, which resulted in no significant differences in the previous study, now caused significant differences in effort for processing the noun between the two verb types. This strongly suggests a significant influence of non-linguistic context on lexical expectations and surprisal-based processing effort. These results bear evidence that listeners map linguistic and visual information, thus making the nouns more predictable, less surprising and crucially easier to process. In other words, anticipatory eye movements patterned nicely with results from the ICA. Whether this correlation is significant will be assessed statistically at a later point in the context of *Experiment 4*.

Further, the noun *“ice cream”* was equally hard to process in both verb conditions, although a clearly favored competitor (*water*) was present in the constraining verb condition.

Anticipation, as reflected by the anticipatory eye movements, on the other hand only elicited a non-significant trend for differences in processing effort in the verb region. That is, the unconstraining verbs, carrying less information in the visual context, were slightly easier to process, compared to the constraining ones. This trend could be attributable to differences in the verbs’ nature in the visual context. That is, the lack of a constraint of verbs such as “order” could cause the listener to put less effort into mapping linguistic to visual information upon encountering the verb, as less information can be gained from this process (see also, e.g., Maess et al., 2016). Alternatively, it could be attributable to the listener’s reduction of visual uncertainty, that is, the exclusion of objects from the display not matching the constraining verb.

The subsequent VWP experiment was designed to specifically quantify the effect of visual context on processing effort and surprisal in the absence of any linguistic variation, i.e., the impact of visual information on expectations and processing effort of a spoken word in contexts with identical linguistic surprisal. It further observed whether the non-significant trend for processing differences on the verb was indeed linked to the reduction of visual uncertainty, rather than to the nature of the verb itself.

Finally, a comparison of the two ICA studies with and without visual context (*Experiment 2* vs. *Experiment 3*) showed a significant overall increase of processing effort in the presence of additional visual information which had to be processed *simultaneously*. We interpret this result as evidence for the direct link between processing effort, as assessed by the ICA, and the amount of information presented, i.e., ‘effort’ is interpreted as the effort of actual information acquisition.

## Experiment 4: Entropy Reduction

### Visual Stimuli Validation

A pre-test assessed whether each piece of clip art used indeed matched the constraint of the verb it was intended to be presented with in the experiment. All clip art was presented in two randomized lists in an online form. Forty people participated and were asked to spontaneously decide whether or not an *object* was *“verb-able,”* by ticking a box stating either “yes” or “no.” All experimental items used in the online studies showed objects that paired well with the verb they were presented with (>90% correct answers per item).

### Methods

The last experiment eliminated any linguistic variation to observe if the surprisal of a word, as modulated by the visual referential context alone, can predict our pupillometric measure of processing effort. This would strongly suggest that surprisal is situated and indexed by the ICA (See also Supplementary Materials for an elaboration of how situated surprisal could be conceived of in more formal terms).

We hence used the same linguistic stimuli across all conditions, featuring only constraining verbs (*spill*) and nouns with high thematic fit (*water*), each presented in four visual contexts where the number of displayed objects matching the verb constraint was manipulated (e.g., 0, 1, 3, or 4 “spillable” objects). This design implied that the same verb reduced visual uncertainty to different degrees between the conditions. Further, target word surprisal only differed when visual information was processed in combination with the sentence, as the linguistic surprisal itself was identical across conditions.

**FIGURE 6:**
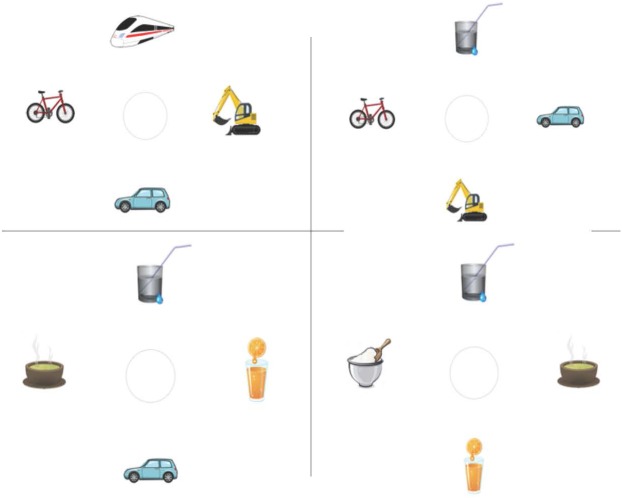
Example stimuli for *Experiment 4.* From left to right and top to bottom: zero, one, three and four possible targets, given the sentence *“The man spills soon the water.”*

**FIGURE 7:**
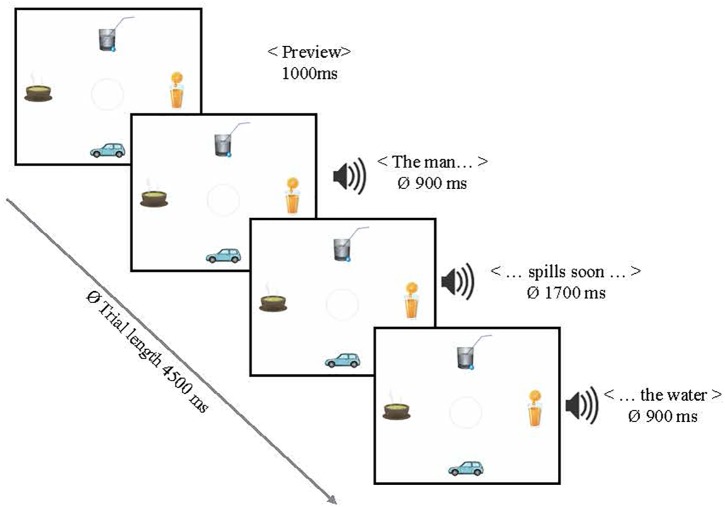
A trial example or *Experiment 4.* The example scene shows three possible target referents.

The 20 highly constraining verbs from the previous studies (followed by the *more plausible* object noun), plus 20 additional new sentences of the same type (in order to increase power), were used in this experiment. In sum, 40 item and 40 filler sentences were combined with the visual displays (for all items see 4 and 5) in such a way that all four conditions of a display (160 in total) shared one sentence. Visual scenes were adapted in the sense that the number of instantiations of a category selected by the verb, i.e., the potential referents, differed. None, one, three, or all four of the objects shown in a scene could be target referents matching a verb (see Figure 6, from left to right and top to bottom). The scenes were counterbalanced between two items in such a way that, for example, a *zero targets* condition picture for one item served as a *four targets* condition in another item. Positions of targets, competitors and distractors in the scenes were rotated. Filler trials introduced variation in terms of the number of categories displayed (i.e., edible, wearable, or drivable objects, etc.). All sentences were presented auditorily and always together (i.e., after a preview time) with the visual displays (see Figure 7).

The identical task and presentation mode were used as in *Experiment 3*. Thirty-two native speakers of German (all students of Saarland University), aged between 18 and 32 years (*M* = 24.56), who had not participated in any of the previous experiments, were tested under informed consent and monetarily reimbursed. A total of 160 visual displays were paired with 40 sentences and split into 4 lists using a Latin square design. Each participant heard each sentence in only one visual condition.

### Predictions

If target word processing effort was significantly influenced by visual context variation, we expected facilitated noun processing in conditions where fewer potential target options are shown (**1** and **3**). In conditions **0** and **4**, on the other hand, no specific expectations about the target could be made in advance. The noun was hence expected to be less predictable, more surprising, and as a result, more effortful to process.

The Entropy Reduction Hypothesis (Hale, 2003) suggests that the more a word reduces uncertainty about the subsequent input, the more difficult it is to process that word. In the case of the mismatch condition **0**, where the verb does not match *any* of the objects displayed, and hence obstructs a matching of the linguistic and visual input string, a major increase in ICA could be possible. That is, although the scene is not helpful and reduces no entropy in this case, we expected the ICA to possibly reflect the detection of a mismatch. If the previously observed non-significant trend for processing differences on the verb was indeed attributable to differences in the verb’s informativity with respect to the reduction of uncertainty in the visual scene, we expected higher processing effort as the verb is more informative, that is, more constraining in its visual context.

### Results

#### Eye Movement Data

**FIGURE 8:**
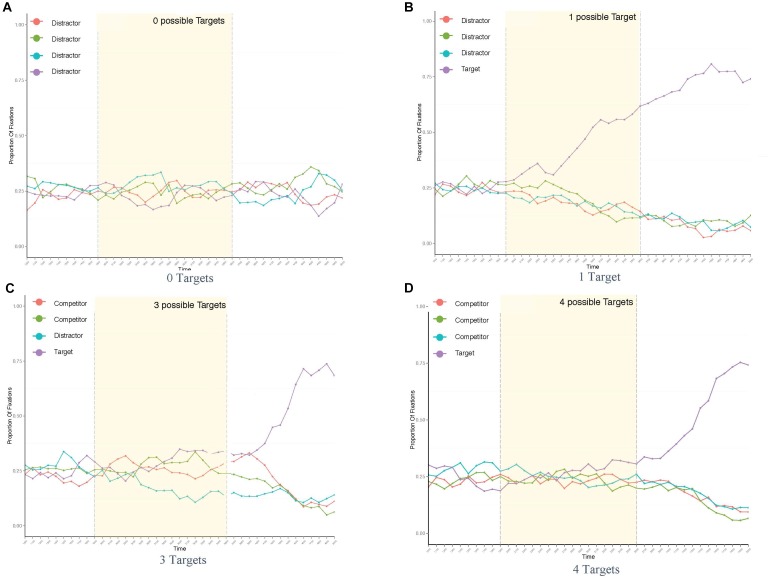
Proportion of fixations across trial length in all conditions of *Experiment 4*: with 0 targets **(A)**, 1 displayed target **(B)**, 3 targets **(C)**, and 4 targets **(D)**.

Overall fixation distribution across an averaged trial in all conditions is plotted for presentation purposes in Figure 8. The distributions reveal an increase in fixations toward objects matching the verb from the onset of the verb onward (left dashed line) when either one or three objects matched the verb constraint, i.e., when the visual scene allowed for more specific expectations about potential target nouns. This indicates a discrimination between those objects that matched the verb and those that did not. No increase in fixations was found when the context did not allow for specific anticipations (conditions **0** and **4**).

Inferential statistics on new inspections between conditions again assessed the probabilities of verb-driven attention shifts toward objects matching the verb.

Analogous to the fixation distribution, new inspections on the verb revealed a significant increase of attention shifts toward the object corresponding to the target noun upon hearing the verb as fewer competitors are shown, i.e., in condition **1** (*M* = 0.21, *SD* = 0.41), compared to **3** (*M* = 0.17, *SD* = 0.38) (β = -0.221, *SE* = 0.099, *z* = -2.21, *p* < 0.05) and to **4** (*M* = 0.16, *SD* = 0.36) (β = -0.293, *SE* = 0.099, *z* = -2.97, *p* < 0.01). In the noun region, listeners were significantly more likely to inspect the mentioned object compared to any other object in the display.

Thus, we again replicated verb-driven anticipatory eye movements in our setup, even when more than one possible target object was displayed. This hints at more (when one object matched the verb) or less (when three objects matched the verb) specific anticipation of the target noun. Whether these anticipations alter surprisal and processing effort either on the verb or on the target noun was assessed by the simultaneously obtained ICA values.

#### Index of Cognitive Activity

**FIGURE 9:**
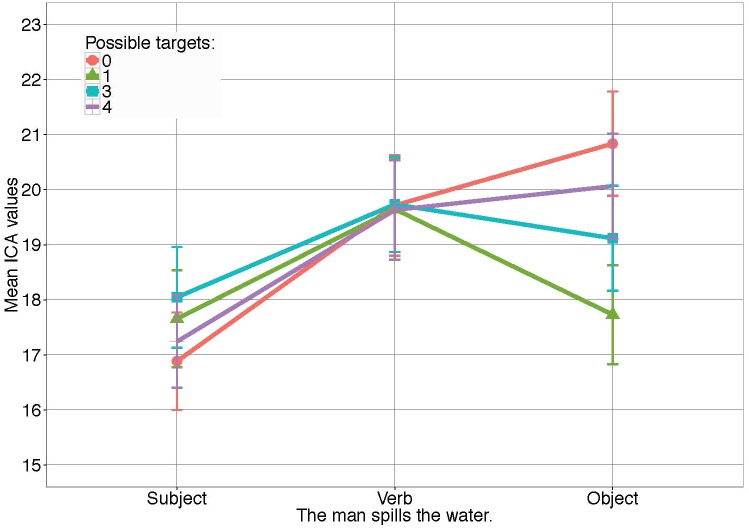
ICA results for *Experiment 4* in all conditions. Error bars reflect 95% confidence intervals (CI). For the models see Supplementary Materials.

Figure 9 shows how ICA events, that is, the number of effort related pupil changes (ICA events) on the verb is similar, while differences between conditions appear on the noun. More specifically, noun processing was facilitated when fewer competitors were displayed, making the noun less surprising.

The obtained ICA values within the pre-defined time windows of interest were treated as count variable and used as the basic dependent variable in generalized mixed effects models of poisson type. Both time windows for ICA analysis were non-overlapping and 600 ms in length, starting from the middle of the critical word’s duration, as previously established for this measure (see e.g., Sekicki and Staudte, 2018). Differences between the conditions (that is, 0 versus 3, 3 versus 4 and 3 versus 1 competitor(s), e.g., “spillable” objects displayed) were contrast coded and entered into the model as fixed factors.

In line with the plot, analysis of the verb window revealed no significant differences between the conditions. This even holds for the linguistic–visual mismatch condition 0, suggesting that anticipatory eye movements, although verb driven, do not elicit differences in surprisal and processing effort on the verb itself. Consequently, the trend observed in the previous experiment was not confirmed. An additional time window of 600 ms length starting from trial onset was analyzed in order to observe possible effects of grouping of the displayed objects prior to hearing the verb, that is, as soon as participants perceive the visual display. No significant differences were found in this region.

Comparisons between conditions in the noun window, however, showed a significant processing facilitation (i.e., lower ICA values) if three competitors were shown (*M* = 19.37, *SD* = 8.17), compared to the unhelpful condition 0, where none of the objects shown were potential referents (*M* = 20.90, *SD* = 8.12) (β = -0.08, *SE* = 0.03, *z* = -2.40, *p* < 0.05). Further, processing of *the water* took significantly less effort when the target object was most predictable, that is, in the presence of only one competitor (*M* = 17.40, *SD* = 7.79), compared to when three competitors were displayed (β = -0.11, *SE* = 0.04, *z* = -2.57, *p* < 0.05). Differences in processing effort between condition 3 and 4 (*M* = 20.13, *SD* = 8.45) did not reach significance. This shows a direct effect of multimodal information on target word surprisal and, linked to that, on the effort needed to process the noun.

#### Correlating Anticipatory Eye Movements With the ICA

Increased ICA values on the noun can be caused by higher surprisal of a noun in the presence of more competitors, or by effort attributable to corrections being made if the listener had predicted one of the competitors to be the target. We hence additionally tested whether the presence of anticipatory glances toward target objects could indeed predict lower ICA values on the noun due to the word already being expected.

The mean number of ICA events differed between trials in which participants directed anticipatory glances toward the target referents as they heard the verb (*M* = 16.8) and trials in which they did not (*M* = 17.6), hinting at a possible correlation between anticipatory looking and processing effort on the noun (although fewer trials were recorded in which no anticipatory eye movements were found). Including the binary variable of target inspections during the verb window in the model resulted in a significantly higher model fit [χ^2^(1) = 7, 25, *p* < 0.05]. The model revealed a main effect of anticipatory target glances on ICA in the noun window (*p* < 0.05), suggesting that their presence during the verb window predicts ICA values on the subsequent noun. We hence propose that differences in the noun’s processing effort are indeed attributable to the predictability of the word in its multimodal context, rather than to corrections of wrong predictions (for a more detailed discussion, see General Summary and Conclusion).

### Discussion

Our results replicated verb-driven anticipatory eye movements toward matching objects (as previously found in many other studies) even in conditions with more than one possible target object. This strongly suggests that listeners exploited the visual information in combination with the verb to anticipate the noun with more or less certainty.

The simultaneously assessed ICA, however, did not differ on the verb. That is, although eye movements showed clear patterns of anticipation, *spill* took equal effort to process, no matter how many competitors were displayed. This was even true for the linguistic-visual mismatch condition 0. We hence propose, that listeners shifted attention toward possible target objects based on the verb information, as indicated by the eye movements, but possibly refrained from deciding on an ultimate exclusion of distractors. It is also possible that a reduction of visual uncertainty (as reflected by different eye movement patterns) does not induce additional effort, or that the ICA is not sensitive toward this sort of effort. Results from Maess et al. (2016) might support the latter. However, their design relied on strong differences in verb constraint, with one verb eliciting a clear lexical expectation, e.g., for the word “orchestra” after “conducts” (“dirigiert”), versus the lack of such a clear expectation after “leads” (“leitet”). Thus, their results cannot be easily compared with our manipulation of visual uncertainty.

The ICA values obtained in the subsequent noun window, however, differed between conditions **0**, **3** and **1**, strongly suggesting that visual context information directly affected the surprisal and processing effort for the target word. Conditions **3** and **4** did not differ significantly from each other. These effects can be interpreted as being analogous to the number of competitors, that is, the probability of a target object in the visual display to correspond to the actual target word coming up: If only one possible target was shown, the noun itself was least surprising and easiest to process, as the displayed object would correspond to the noun with 100% certainty (condition **1**). The same noun was more surprising when three competitors were shown and this correspondence was only 33% certain (condition **3**). The lack of significant differences between conditions **3** and **4** might then be attributable to either a lack of power, or three and four being too similar to result in measurable differences. Alternatively, the results may reflect a rough decision on whether one, many, or none of the objects matched the verb, while no further evaluation of the context was performed by the participants.

The mismatch between the visual and linguistic information in condition **0** caused a similarly high processing effort on the verb or the noun, compared to condition four, although no additional information was given to prepare the listener for the upcoming words. A possible explanation for this could be that the mismatch between two different modalities is not reflected in the ICA. It is conceivable that the detection of a modality-mismatch elicits effort of a different quality, that is, effort with respect to something distinct from information processing. An electro-physiological measure might help to tease this apart.

In sum, we interpret these findings as robust evidence for the significant effect of visual context information on (linguistically identical) surprisal-based processing effort for the target word.

## General Summary and Conclusion

In four experiments deploying behavioral and pupillometric measures, we closely examined the effect of visual context information on target word predictability and the associated situated surprisal as well as processing effort.

Apart from replicating anticipatory eye movements to objects depicting possible target referents in all our VWP studies – even in contexts in which three or four possible targets were displayed – we found reliable evidence for the immediate effect of visual information on overall processing effort, as well as on expectations formed about the target word and, consequently, its surprisal-based processing effort.

While our finding that the purely linguistic context in our initial experiments did not result in specific predictions beyond semantic categories (the only effect measured was in reaction to a semantic category violation) initially seemed surprising, there are factors that can account for those results. That is, most studies that find predictability effects in linguistic context (Kutas and Federmeier, 1999; Rayner et al., 2004) usually featured stronger contextual constraints and larger differences in cloze probabilities. In the case of the presented studies, the verb constraint was the only manipulation of predictability and the nouns were of rather low cloze value (even the predictable ones). Smith and Levy (2013), further reported a logarithmic effect of word predictability on reading times. It is quite possible, however, that such effects can only be found in large amounts of (language modeled) data and by calculating average surprisal values.

In general, while the same linguistic stimuli caused a null result in very mildly constraining, purely linguistic contexts, the addition of visual context immediately caused an overall increase in processing effort throughout the trials, as well as differences in surprisal-based processing effort on the target words.

While the process of anticipation – or uncertainty/entropy reduction – itself, as reflected by the eye movement data, did not elicit any differences in processing cost, results from the noun window revealed effects of visual context on word processing. That is, neither the effort of shifting attention to fewer or more objects, nor the exclusion of distractors, showed corresponding effects on the verb. Although the results from the noun window are a result of the reduction of visual uncertainty on the verb, ICA *does not* show any differences here. One reason may be that listeners did not actually *exclude* distractors as options for upcoming nouns, but that they only internally attributed higher saliency to suitable competitor objects. This rather subtle difference would also explain why Maess et al. (2016), unlike us, found effects. In their study, verbs differed strongly in constraint and either allowed for concrete lexical expectations, or did not. Another explanation could be that higher (visual) entropy reduction simply does not require higher processing effort, as is the case for highly surprising words. And lastly, it is also conceivable that surprisal and entropy reduction recruit different processes, and therefore different *kinds* of activity or effort, and that ICA indexes the former but is insensitive to the latter.

Interestingly, not even the mismatch between visual and linguistic input in condition **0** of *Experiment 4*, which would be expected to show a disruptive effect in other measures such as the EEG, elicited higher ICA values. We interpret this lack of an effect as being caused by the insensitivity of the ICA with respect to the sort of effort caused by a mismatch of modalities. Instead, the ICA is very sensitive with respect to effort caused by information processing. A comparison of studies with and without simultaneously presented visual context further showed an overall increase of processing effort as more information is added.

In the noun window, however, *Experiment 3* showed that in a visual context, the more plausible target word *water* was more expected than *ice cream*, when following the constraining verb *spill*. Such effects were found neither in the behavioral measures nor in the ICA of the same stimuli without visual context.

Visual context effects in *Experiment 4* even seemed to be graded in reaction to the given multimodal context. This was despite the absence of any linguistic variation. That is, processing of the same noun was facilitated as fewer objects in the visual scene matched the verb. More precisely, *the water* took least effort to process when only one possible target was shown. The same noun was significantly harder to process as three objects from the scene matched the verb and took the most effort as none of the displayed objects were possible referents. It is conceivable that the differences in processing effort for the nouns are caused by the revision of incorrect expectations, rather than by the actual predictability of the word. In other words, it is possible that with more competitors, participants were more likely to look at a different object than the target noun and that they, thus, had to revise their current interpretation. However, this would mean that listeners commit randomly to one object (possibly for optical reasons) prior to hearing the actual referent noun. Especially in the purely linguistic contexts, our experiments revealed that participants are rather unlikely to make strong predictions without supporting evidence, possibly because they would be inefficient and error prone. One might argue that, the observed pupillary effects are due to an increasing complexity in saccade planning in the case of more, compared to less, competitors in the display. That is, the planning of looks toward four attractive objects is expected to cause more effort, compared to the planning of looks to only one potential target displayed. In this case, the measured effort and the respective differences would be interpreted with respect to saccade planning rather than predictive processing. However, since the ICA is a relatively direct measure, we would expect such differences earlier, namely between the verb and the actual noun, where saccades to target and competitors are planned and actually conducted. At the time of the noun, however, participants always looked at only one object, namely the one corresponding to the noun, with the exception of the mismatch condition **0**, where no matching object was shown. Especially in this condition, we would expect the effort – if related to saccade planning – to differ from condition **4**.

We hence suggest that our results can be interpreted as being graded with respect to the target word’s multi-modal predictability. That is, the probability of an object to come up as target noun in the scene-sentence combinations. Specifically, the probability of the object water to come up as a label in the particular situation of condition **1** was essentially 1.0. At the same time, noun processing required higher processing effort when three competitors were shown, lowering the probability of each of the competitor objects to come up as a noun to 0.33. In this case, we suggest that conditions **3** and **4** not differed significantly due to a lack of power, or, alternatively, due to the difference of only one competitor being too small to elicit significant differences in processing effort for the noun. Alternatively, the results could reflect a rough discrimination between one, many or no objects being possible targets, given the verb information, without any further probabilistic evaluation of the context. The question whether the observed differences are indeed probabilistic, or rather reflect a one-many-nothing discrimination requires additional research.

In sum, we conclude that a word’s surprisal – as modulated by the visual context – predicts our pupillometric (ICA) measures on online processing effort. We provide evidence that surprisal, and its associated processing effort, is hence not determined by the linguistic signal alone, but rather reflects expectations derived online from (at least) the relevant visual environment in which an utterance is processed.

## Data Availability Statement

The dataset(s) supporting the conclusions of this article will be made available upon request.

## Ethics Statement

This study was carried out in accordance with the recommendations of the American Psychological Association, with written informed consent from all subjects. All subjects gave written informed consent in accordance with the Declaration of Helsinki. The protocol was approved by the ethics committee by the Deutsche Gesellschaft für Sprache (DGfS).

## Author Contributions

CA, MiS, and MaS initiated and framed the research questions. CA and MiS designed, conducted and performed the statistical analysis for Experiments 1, 2, and 3. CA designed, conducted and performed the statistical analysis for Experiment 4. All authors edited, read and approved the final manuscript.

## Conflict of Interest Statement

The authors declare that the research was conducted in the absence of any commercial or financial relationships that could be construed as a potential conflict of interest.

## Footnotes

1 The unusual word order is the result of a word-for-word translation from German.

2 For validation of the categories, see Section “Validation”.

3 (DeReKo corpus size > 28 billion words. Source: Korpusbasierte Wortgrundformenliste DeReWo,2012, v-ww-bll-320000g-2012-12-31-1.0, http://www.ids-mannheim.de/derewo).

4 Note that the cloze task was only used to assess the strength of verb constraints. All object nouns were filtered from DeReWo, rather than being picked according to the cloze results.

5 i.e., a measure that is not affected in the temporal dimension (as reading or reaction times are) but rather in energy or amplitude (like ERPs).

6 Neither were noun frequencies included as a covariate, since head noun frequencies were controlled for within each item. Frequencies were assessed using the word lists DeReWo, http://www.ids-mannheim.de/derewo.
