# Supplementary material for: The Influence of Visual Uncertainty on Word Surprisal and Processing Effort
Source: Front Psychol. 2018 Dec 14;9:2387. doi: 10.3389/fpsyg.2018.02387 (PMC6302025; doi:10.3389/fpsyg.2018.02387)
Supplement: Supplementary file 1 [file Table_1.PDF]

frontiers

Supplementary Material: Article Title

1 SUPPLEMENTARY DATA

2 FORMALISATION OF VISUALLY INSPIRED SURPRISAL

In order to account for the observed influence of visual information on information processing, we suggest to determine surprisal from the relative probabilities of *only* the co-present objects as follows:

We define λ as the sum of all linguistically-derived probabilities of all potentially upcoming words, resulting in 1. In the case of *co-present* objects that would match the verbal constraint, however, those take precedence as upcoming nouns over other possible *non-present* continuations and compose the new set of alternatives. Thus, λ reduces to the sum over (visually relevant) nouns:

$$\lambda := \sum_{w_i \in PotentialTargetObjects} p(w_i | w_1, ..., w_{i-1})$$

By dividing the probability of a single noun w_i by λ, the probability of w_i will be scaled to the available set of alternatives while preserving the relative probabilities. We thus propose visually-informed surprisal to be:

$$S(w_i) = -\log_2 \frac{p(w_i | w_1, w_2, ...w_{i-1})}{\lambda}$$

Notice that if no visual context is given, the sum of the probabilities for all possible nouns would be 1, resulting in the original linguistic surprisal value.

In order to test the reliability of the proposed formula extension, we applied it to surprisal values calculated based on language-model-derived probabilities of all competitors in a scene, given the prior verb context. Probability distributions over instances filling the thematic roles in all our items (object, given the prior predicate, both translated to English) were calculated using the language model described in **?**. This neural network model was especially suitable for our purposes, since it was specifically trained to represent event-relevant context and predict human thematic fit ratings, making it possible to calculate event-level surprisal from the model's probabilities.

Applying the extended surprisal formula to the model values yielded the following results (averaged over each condition):

Surprisal on the target noun (plausible)[6], given the constraining verb (e.g. *spill the water*) in a purely linguistic context:
$S(noun)_{ling.only} = -\log_2(0.008)$
$S(noun)_{ling.only} = 6.96$

[6] Probabilities of all nouns corresponding to depicted competitors, given the preceding verb (e.g., SPILL_WATER, SPILL_JUICE, etc.) were calculated using the Language Model described in **?**.

Surprisal on the target noun (plausible), given the constraining verb (e.g., *spill the water*) in a visual context featuring one possible target object (condition 1 in ***Experiment 3***):

$S(noun)_{cond.1} = -\log_2 \frac{0.008}{0.008}$

$S(noun)_{cond.1} = 0$

Surprisal on the target noun (plausible), given the constraining verb (e.g., *spill the water*) in a visual context featuring three possible target objects (condition 3 in ***Experiment 3***):

$S(noun)_{cond.3} = -\log_2 \frac{0.008}{(0.008)+(0.005)+(0.01)}$

$S(noun)_{cond.3} = 1.51$

Surprisal on the target noun (plausible), given the constraining verb (e.g., *spill the water*) in a visual context featuring four possible target objects (condition 4 in ***Experiment 3***):

$S(noun)_{cond.4} = -\log_2 \frac{0.008}{(0.008)+(0.005)+(0.01)+(0.002)}$

$S(noun)_{cond.4} = 1.61$

Note, however, that the absolute ICA values in the visual studies were significantly higher overall, compared to the language-only ***Experiment 1***, most likely due to the increased effort needed to simultaneously process information from several modalities. Hence, visually-informed surprisal alone cannot account for all kinds of cognitive load involved in the processing of multimodal information.

Table S1 shows the average ICA (as measured in each condition in ***Experiment 4***), as well as linguistic surprisal (as calculated from LM-derived probabilities) and surprisal values according to the proposed formula, along with a representative example item from the stimuli set. While the purely linguistic surprisal values cannot describe the changes in ICA values in response to the different probability profiles of the varying visual scenes, "visually informed" surprisal can.

Table S1. Representative example item with average values for classical linguistic surprisal, visually informed surprisal and the corresponding ICA values from ***Experiment 3***.

Item	Condition target options	Surprisal[7] (LM-derived prob.)	Surprisal *visually informed*	ICA
	1	6.96	0.0	17.6
(1) *The man spills soon the water*	3	6.96	1.51	19.2
	4	6.96	1.61	20.1

[7] Condition 0 is not shown in the table as the complex processing of the mismatch between visual and verb information can not be formalised without further experimental examination of this process.

3 MODELS

*Models used for ICA analysis in **Experiment 2**:*

Analysis for the verb window:

ICA on Verb $\sim$ VerbCondition + Scaled Verb Duration +

$(1 + Verb||Participant)$ +

$(1 + Verb||Item)$, family=poisson.

Analysis for the noun window:

ICA on Noun $\sim$ Verb-Object Interaction + Verb + Object +

$(1 + Verb + Object + Verb - ObjectInteraction||Participant)$ +

$(1 + Verb + Object + Verb - ObjectInteraction||Item)$, family=poisson

*Models used for ICA analysis in **Experiment 3**:*

ICA on Verb/Noun $\sim$ Verb-Object Interaction + Verb + Object +

$(1 + Verb + Object|Participant)$ +

$(1 + Verb + Object|Item)$, family=poisson

*Models used for ICA comparison between studies **Experiment 2** and **Experiment 3**:*

ICA on Noun/Verb $\sim$ Experiment + Verb-Object interaction + Verb + Object +

Verb-Experiment interaction + Object-Experiment interaction +

Verb-Object-Experiment interaction +

$(1 + Verb + Object + V - Objectinteraction||Participant)$ +

$(1 + Verb + Object + Verb - Objectinteraction||Item)$, family=poisson

*Models used for ICA analysis in **Experiment 4**:*

ICA on Noun/Verb $\sim$ + target number conditions +

$(1 + TargetNumberConditions|Participant)$ +

$(1 + TargetNumberConditions|Item)$, family=poisson

4 LINGUISTIC STIMULI

Table S2: *Linguistic stimuli for studies* ***Experiment 1*** *and* ***Experiment 2.***
The Table shows all linguistic items in each condition: Each item had a constraining and an unconstraining verb condition (see columns 3&4), each paired with two different objects (see columns 6&7). Column 1 indicates the ID of the item itself and the scenes which were shown together with the sentence in the VWP studies. The complete set of all visual stimuli is listed in Section 5

Item Nr.		Verb	Verb		Object 1	Object2
		constraining	unconstraining		plausible	possible
1	Die Mutter	löffelt	bewertet	gleich	die Suppe	den Kaffee
	The mother	*sups*	*rates*	*soon*	*the soup*	*the coffee*
2	Der Grossvater	verschüttet	bestellt	gleich	das Wasser	das Eis
	The grandfather	*spills*	*orders*	*soon*	*the water*	*the ice cream*
3	Die Schwester	schmilzt	kontrolliert	gleich	die Butter	den Honig
	The sister	*melts*	*checks*	*soon*	*the butter*	*the honey*
4	Die Cousine	montiert	ersetzt	gleich	die Antenne	das Motorrad
	The cousin	*assembles*	*replaces*	*soon*	*the antenna*	*the motorbike*
5	Der Grossvater	kocht	verpackt	gleich	die Kartoffel	die Banane
	The grandfather	*cooks*	*wraps*	*soon*	*the potato*	*the banana*
6	Die Grossmutter	trinkt	testet	gleich	den Kaffee	den Joghurt
	The grandmother	*drinks*	*tests*	*soon*	*the coffee*	*the yoghurt*
7	Der Grossvater	serviert	fotografiert	gleich	das Eis	die Zitrone
	The grandfather	*serves*	*photographs*	*soon*	*the ice cream*	*the lemon*
8	Der Grossvater	isst	nimmt	gleich	das Brot	den Pfeffer
	The grandfather	*eats*	*grabs*	*soon*	*the bread*	*the pepper*
9	Der Cousin	poliert	erblickt	gleich	das Auto	den Zug
	The cousin	*polishes*	*beholds*	*soon*	*the car*	*the train*
10	Der Vater	kühlt	reklamiert	gleich	den Wein	die Waffel
	The father	*cools*	*exchanges*	*soon*	*the wine*	*the waffle*
11	Die Schwester	bestickt	behält	gleich	das Kissen	den Stiefel
	The sister	*embroiders*	*keeps*	*soon*	*the pillow*	*the boot*
12	Die Grossmutter	zuckert	prüft	gleich	den Tee	die Zitrone
	The grandmother	*sweetens*	*checks*	*soon*	*the tea*	*the lemon*
13	Der Mann	fährt	sieht	gleich	das Auto	das Schiff
	The man	*drives*	*sees*	*soon*	*the car*	*the ship*
14	Der Cousin	näht	berührt	gleich	die Jacke	das Sofa
	The cousin	*sews*	*touches*	*soon*	*the jacket*	*the sofa*
15	Die Frau	schneidet	holt	gleich	das Brot	die Pommes
	The woman	*cuts*	*gets*	*soon*	*the bread*	*the fries*
16	Die Mutter	flickt	vergisst	gleich	die Jeans	den Besen
	The mother	*repairs*	*forgets*	*soon*	*the Jeans*	*the broom*
17	Die Frau	bügelt	beschreibt	gleich	das T-Shirt	die Socke
	The woman	*irons*	*describes*	*soon*	*the t-shirt*	*the sock*
18	Die Mutter	strickt	bekommt	gleich	den Schal	die Decke

Table S2: *Linguistic stimuli for studies* ***Experiment 1*** *and* ***Experiment 2.***
The Table shows all linguistic items in each condition: Each item had a constraining and an unconstraining verb condition (see columns 3&4), each paired with two different objects (see columns 6&7). Column 1 indicates the ID of the item itself and the scenes which were shown together with the sentence in the VWP studies. The complete set of all visual stimuli is listed in Section 5

Item Nr.		Verb<br>constraining	Verb<br>unconstraining		Object 1<br>plausible	Object2<br>possible
	The mother	*knits*	*receives*	*soon*	*the scarf*	*the blanket*
19	Die Frau	erntet	wäscht	gleich	den Apfel	den Reis
	The woman	*harvests*	*washes*	*soon*	*the apple*	*the rice*
20	Der Bruder	repariert	zeichnet	gleich	den Laptop	das Raumschiff
	The brother	*repairs*	*draws*	*soon*	*the laptop*	*the space ship*

Table S3: *Linguistic stimuli for* ***Experiment 3***.
The Table shows all linguistic items used. Each item had four different corresponding scenes, shown in the figure below this table. Column 1 indicates the ID of the item itself and the scenes which were shown together with the sentences. The asterisk indicates which items have also been used in the other experiments.

Item Nr.		Verb		Object
1*	Die Frau	löffelt	gleich	die Suppe
	The woman	*sups*	*soon*	*the soup*
2*	Die Frau	verschüttet	gleich	das Wasser
	The woman	*spills*	*soon*	*the water*
3*	Die Frau	packt	gleich	den Koffer
	The woman	*packs*	*soon*	*the suitcase*
4*	Die Frau	fährt	gleich	das Auto
	The woman	*drives*	*soon*	*the car*
5*	Die Frau	entsaftet	gleich	die Orange
	The woman	*juices*	*soon*	*the orange*
6*	Der Mann	montiert	gleich	die Antenne
	The man	*assembles*	*soon*	*the antenna*
7*	Die Frau	bügelt	gleich	das T-Shirt
	The woman	*irons*	*soon*	*the t-shirt*
8*	Der Mann	trinkt	gleich	den Kaffee
	The man	*drinks*	*soon*	*the coffee*
9*	Die Frau	verbiegt	gleich	die Büroklammer
	The woman	*bends*	*soon*	*the paper clip*
10*	Der Mann	würzt	gleich	den Salat
	The man	*seasons*	*soon*	*the salad*
11*	Die Frau	erntet	gleich	den Apfel
	The woman	*harvests*	*soon*	*the apple*
12*	Der Mann	poliert	gleich	das Auto
	The man	*polishes*	*soon*	*the car*

Table S3: *Linguistic stimuli for* ***Experiment 3***.
The Table shows all linguistic items used. Each item had four different corresponding scenes, shown in the figure below this table. Column 1 indicates the ID of the item itself and the scenes which were shown together with the sentences. The asterisk indicates which items have also been used in the other experiments.

Item Nr.		Verb		Object
13*	Der Mann	pflanzt	gleich	die Blume
	The man	*plants*	*soon*	*the flower*
14*	Die Frau	bestickt	gleich	das Kissen
	The woman	*embroiders*	*soon*	*the pillow*
15*	Die Frau	bäckt	gleich	den Keks
	The woman	*bakes*	*soon*	*the cookie*
16*	Die Frau	isst	gleich	den Burrito
	The woman	*eats*	*soon*	*the burrito*
17	Der Mann	knackt	gleich	den Tresor
	The man	*cracks*	*soon*	*the safe*
18*	Die Frau	näht	gleich	die Jacke
	The woman	*sews*	*soon*	*the jacket*
19	Der Mann	spült	gleich	den Topf
	The man	*washes*	*soon*	*the pot*
20	Der Mann	schält	gleich	die Zwiebel
	The man	*peels*	*soon*	*the onion*
21	Der Mann	raucht	gleich	die Zigarre
	The man	*smokes*	*soon*	*the cigar*
22	Die Frau	grillt	gleich	die Wurst
	The woman	*barbecues*	*soon*	*the sausage*
23	Die Frau	lenkt	gleich	den Hubschrauber
	The woman	*steers*	*soon*	*the helicopter*
24	Die Frau	stimmt	gleich	das Klavier
	The woman	*tunes*	*soon*	*the piano*
25	Die Frau	belegt	gleich	die Pizza
	The woman	*tops*	*soon*	*the pizza*
26	Die Frau	bindet	gleich	den Schuh
	The woman	*ties*	*soon*	*the shoe*
27	Die Frau	giesst	gleich	die Rose
	The woman	*waters*	*soon*	*the rose*
28	Der Mann	kocht	gleich	die Kartoffel
	The man	*cooks*	*soon*	*the potato*
29*	Die Frau	schneidet	gleich	das Brot
	The woman	*cuts*	*soon*	*the bread*
30	Die Frau	flickt	gleich	die Jeans
	The woman	*repairs*	*soon*	*the jeans*
31	Der Mann	liest	gleich	die Zeitung
	The man	*reads*	*soon*	*the newspaper*

Table S3: *Linguistic stimuli for* ***Experiment 3***.
The Table shows all linguistic items used. Each item had four different corresponding scenes, shown in the figure below this table. Column 1 indicates the ID of the item itself and the scenes which were shown together with the sentences. The asterisk indicates which items have also been used in the other experiments.

Item Nr.		Verb		Object
32	Der Mann	zuckert	gleich	den Tee
	The man	*sweetens*	*soon*	*the tea*
33	Der Mann	brät	gleich	das Fleisch
	The man	*cooks*	*soon*	*the meat*
34*	Der Mann	repariert	gleich	den Laptop
	The man	*repairs*	*soon*	*the laptop*
35	Der Mann	streicht	gleich	den Stuhl
	The man	*paints*	*soon*	*the chair*
36	Der Mann	serviert	gleich	das Eis
	The man	*serves*	*soon*	*the ice cream*
37	Der Mann	rührt	gleich	den Joghurt
	The man	*stirs*	*soon*	*the yoghurt*
38	Der Mann	spitzt	gleich	den Buntstift
	The man	*sharpens*	*soon*	*the crayon*
39*	Die Frau	strickt	gleich	den Schal
	The woman	*knits*	*soon*	*the scarf*
40	Die Frau	buttert	gleich	die Brezel
	The woman	*butters*	*soon*	*the pretzel*

5 VISUAL STIMULI

Figure S1: *Visual stimuli for study* ***Study 2***. All item scenes [1]used in ***Study1*** and ***Study 2***. Column 1 in the above given table indicates the matching linguistic stimuli presented in combination with the pictures.

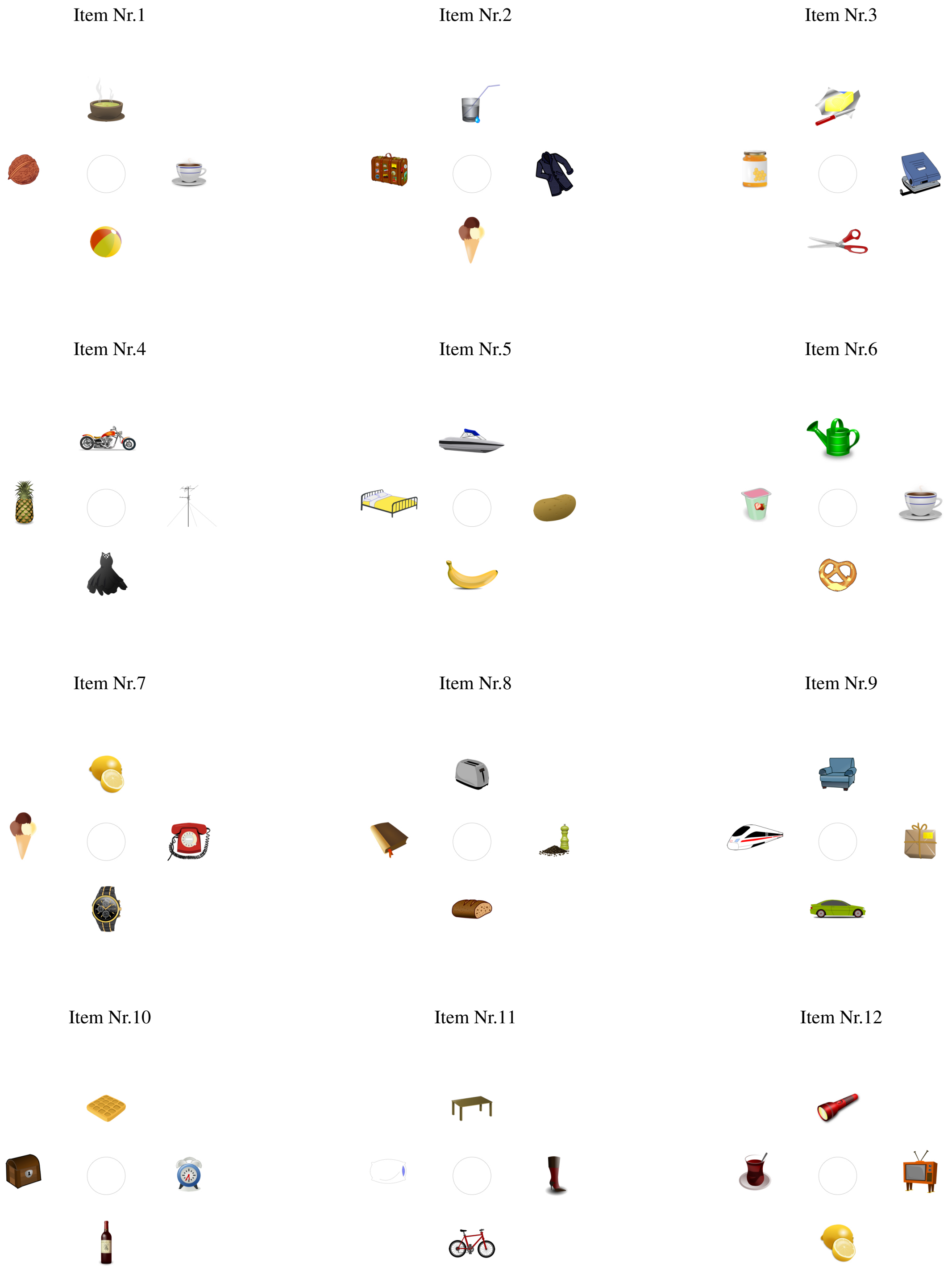

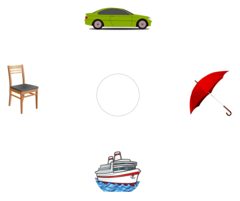

Item Nr.13

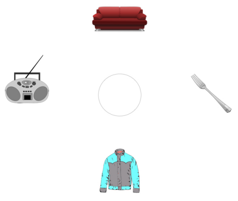

Item Nr.14

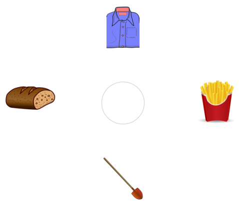

Item Nr.15

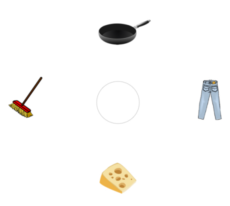

Item Nr.16

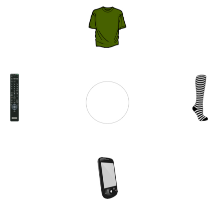

Item Nr.17

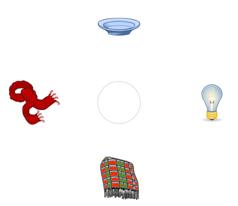

Item Nr.18

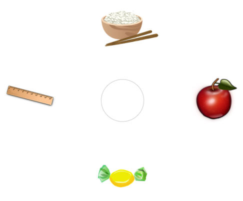

Item Nr.19

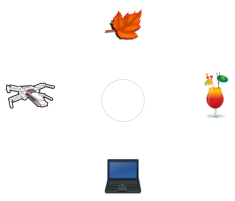

Item Nr.20

[1] Each scene was compiled of four clip arts. All clip arts have been retrieved from: http://www.clipartkid.com, http://clipart-library.com, http://www.clipartpanda.com, https://openclipart.org, https://pixabay.com

Figure S2: *Visual stimuli for **Study 3***. All item scenes [2]in all conditions used.Each scene has four conditions: Showing 0, 1, 3 or 4 possible targets. All four conditions of a scene correspondet to the same linguistic stimulus. Column 1 in the above given table indicates the matching linguistic stimuli presented in combination with the pictures.

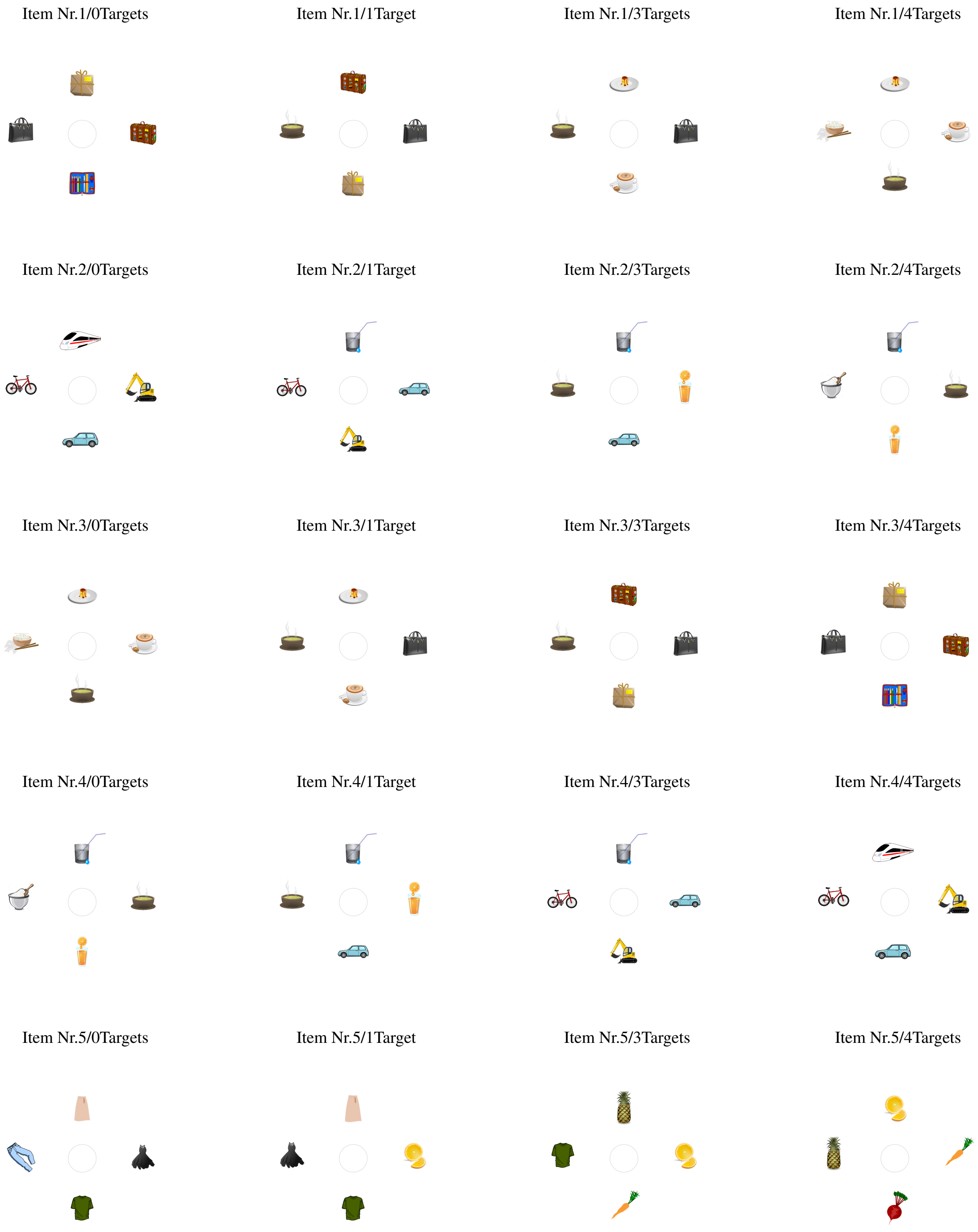

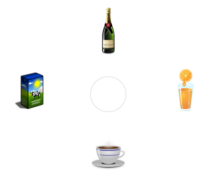
Item Nr.6/0Targets

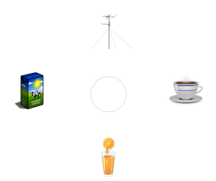
Item Nr.6/1Target

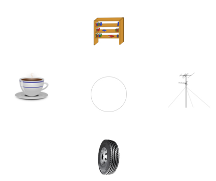
Item Nr.6/3Targets

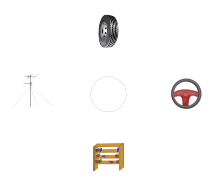
Item Nr.6/4Targets

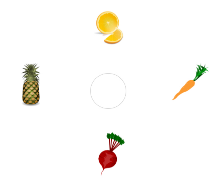
Item Nr.7/0Targets

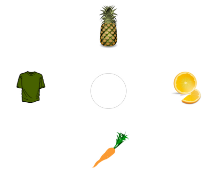
Item Nr.7/1Target

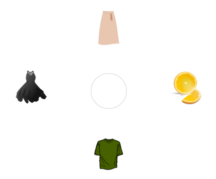
Item Nr.7/3Targets

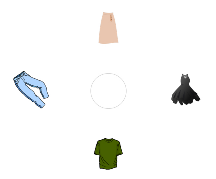
Item Nr.7/4Targets

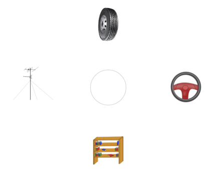
Item Nr.8/0Targets

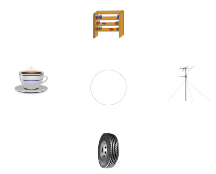
Item Nr.8/1Target

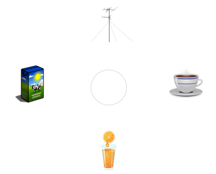
Item Nr.8/3Targets

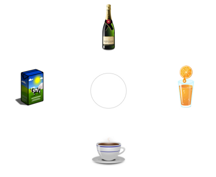
Item Nr.8/4Targets

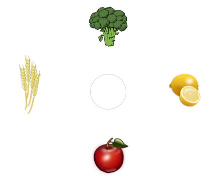
Item Nr.9/0Targets

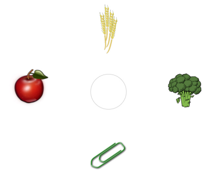
Item Nr.9/1Target

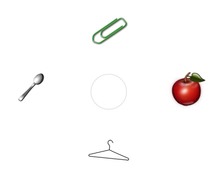
Item Nr.9/3Targets

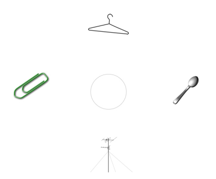
Item Nr.9/4Targets

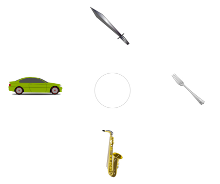
Item Nr.10/0Targets

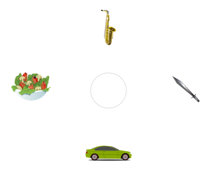
Item Nr.10/1Target

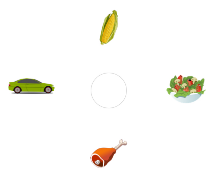
Item Nr.10/3Targets

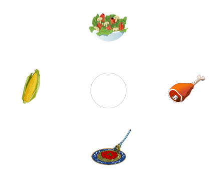
Item Nr.10/4Targets

[2] Each scene was compiled of four clip arts each. All clip arts have been retrieved from: `http://www.clipartkid.com`, `http://clipart-library.com`, `http://www.clipartpanda.com`, `https://openclipart.org`, `https://pixabay.com`

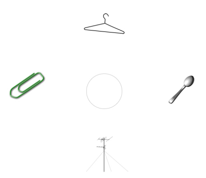
Item Nr.11/0Targets

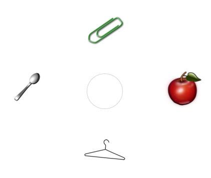
Item Nr.11/1Target

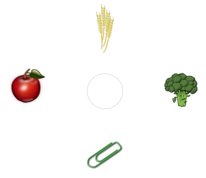
Item Nr.11/3Targets

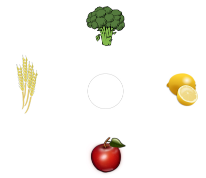
Item Nr.11/4Targets

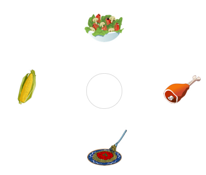
Item Nr.12/0Targets

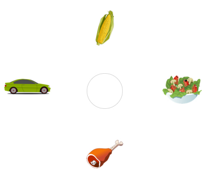
Item Nr.12/1Target

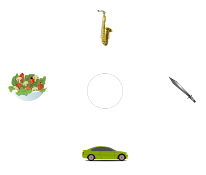
Item Nr.12/3Targets

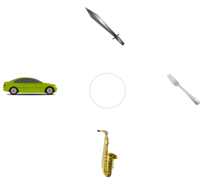
Item Nr.12/4Targets

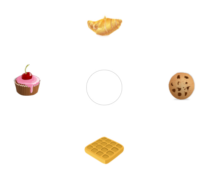
Item Nr.13/0Targets

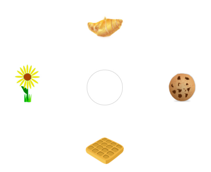
Item Nr.13/1Target

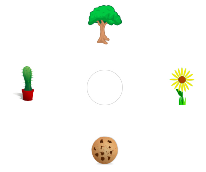
Item Nr.13/3Targets

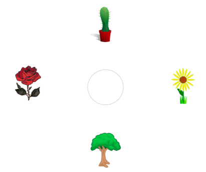
Item Nr.13/4Targets

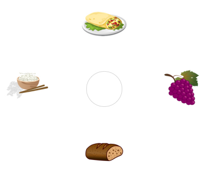
Item Nr.14/0Targets

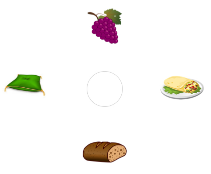
Item Nr.14/1Target

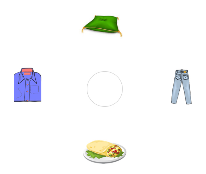
Item Nr.14/3Targets

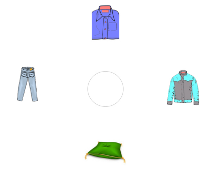
Item Nr.14/4Targets

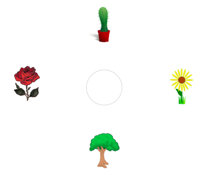
Item Nr.15/0Targets

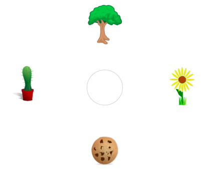
Item Nr.15/1Target

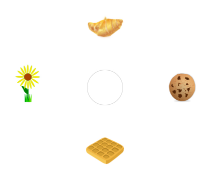
Item Nr.15/3Targets

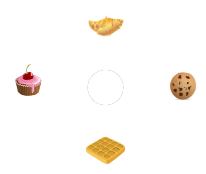
Item Nr.15/4Targets

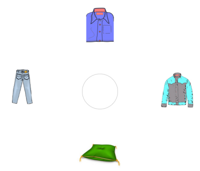
Item Nr.16/0Targets

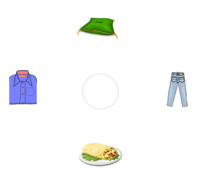
Item Nr.16/1Target

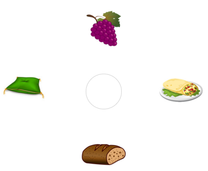
Item Nr.16/3Targets

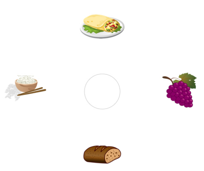
Item Nr.16/4Targets

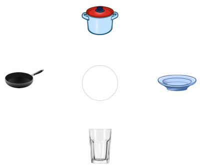
Item Nr.17/0Targets

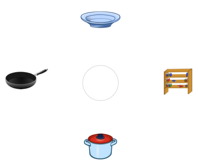
Item Nr.17/1Target

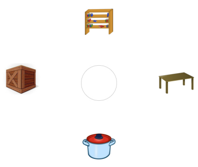
Item Nr.17/3Targets

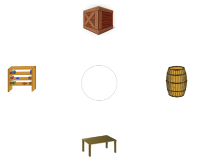
Item Nr.17/4Targets

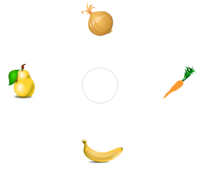
Item Nr.18/0Targets

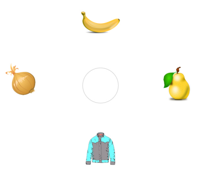
Item Nr.18/1Target

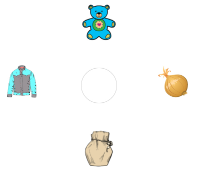
Item Nr.18/3Targets

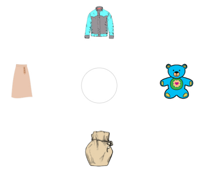
Item Nr.18/4Targets

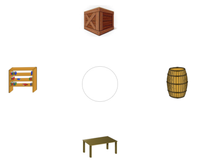
Item Nr.19/0Targets

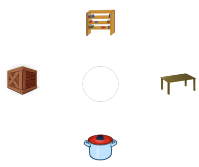
Item Nr.19/1Target

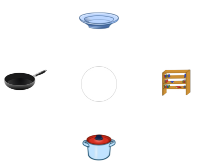
Item Nr.19/3Targets

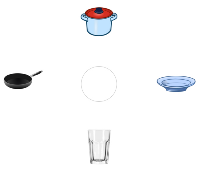
Item Nr.19/4Targets

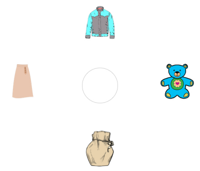
Item Nr.20/0Targets

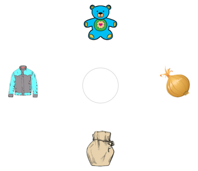
Item Nr.20/1Target

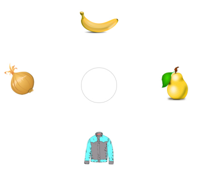
Item Nr.20/3Targets

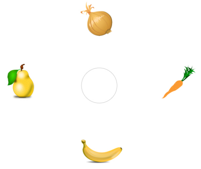
Item Nr.20/4Targets

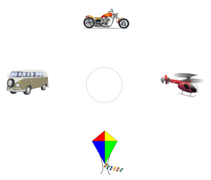
Item Nr.21/0Targets

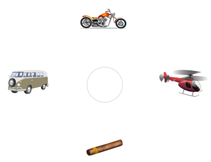
Item Nr.21/1Target

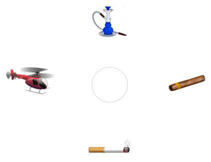
Item Nr.21/3Targets

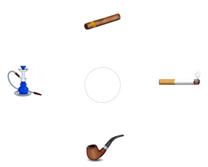
Item Nr.21/4Targets

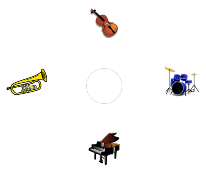
Item Nr.22/0Targets

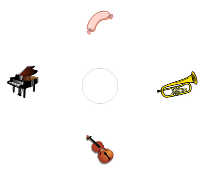
Item Nr.22/1Target

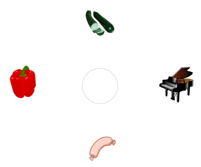
Item Nr.22/3Targets

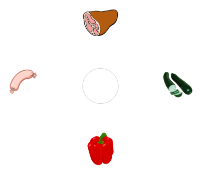
Item Nr.22/4Targets

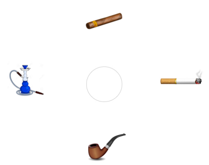
Item Nr.23/0Targets

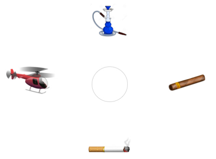
Item Nr.23/1Target

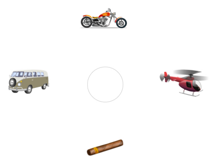
Item Nr.23/3Targets

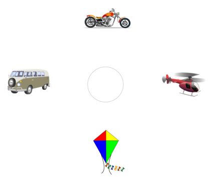
Item Nr.23/4Targets

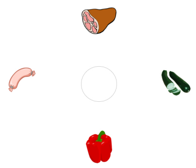
Item Nr.24/0Targets

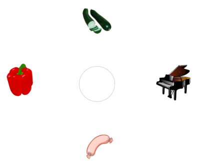
Item Nr.24/1Target

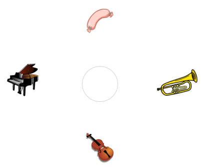
Item Nr.24/3Targets

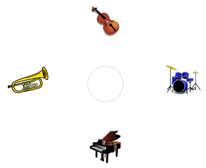
Item Nr.24/4Targets

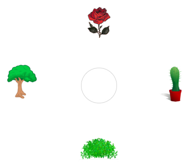
Item Nr.25/0Targets

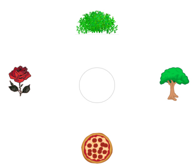
Item Nr.25/1Target

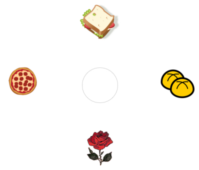
Item Nr.25/3Targets

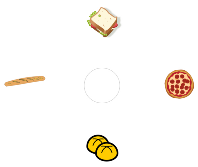
Item Nr.25/4Targets

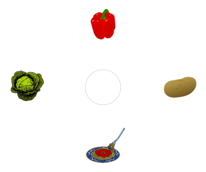
Item Nr.26/0Targets

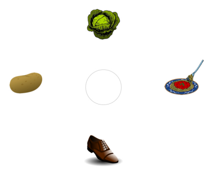
Item Nr.26/1Target

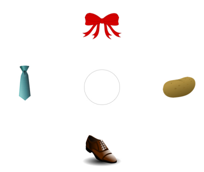
Item Nr.26/3Targets

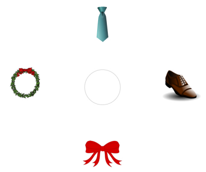
Item Nr.26/4Targets

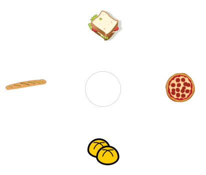
Item Nr.27/0Targets

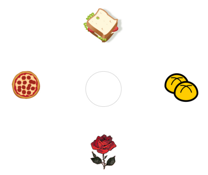
Item Nr.27/1Target

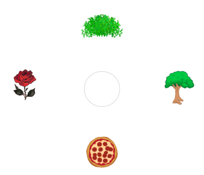
Item Nr.27/3Targets

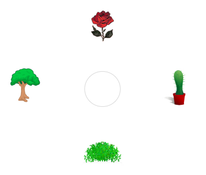
Item Nr.27/4Targets

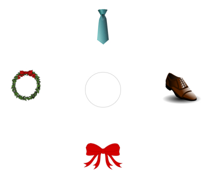
Item Nr.28/0Targets

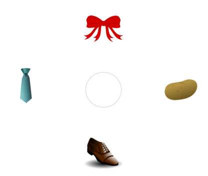
Item Nr.28/1Target

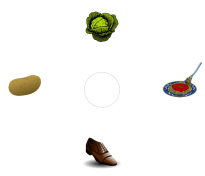
Item Nr.28/3Targets

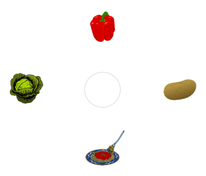
Item Nr.28/4Targets

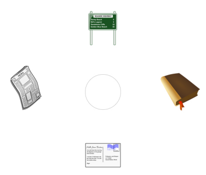
Item Nr.29/0Targets

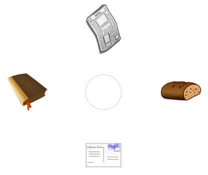
Item Nr.29/1Target

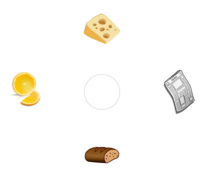
Item Nr.29/3Targets

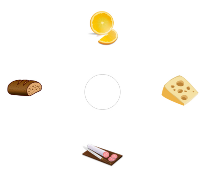
Item Nr.29/4Targets

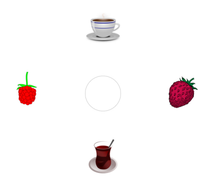
Item Nr.30/0Targets

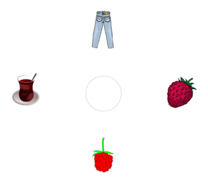
Item Nr.30/1Target

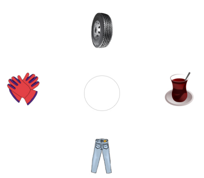
Item Nr.30/3Targets

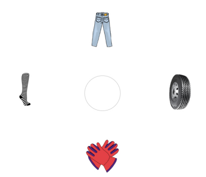
Item Nr.30/4Targets

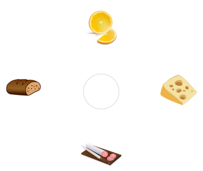

Item Nr.31/0Targets

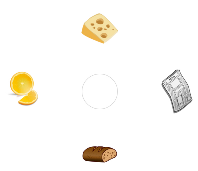

Item Nr.31/1Target

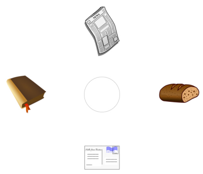

Item Nr.31/3Targets

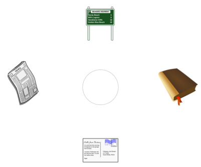

Item Nr.31/4Targets

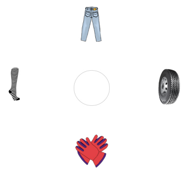

Item Nr.32/0Targets

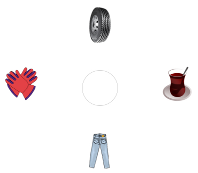

Item Nr.32/1Target

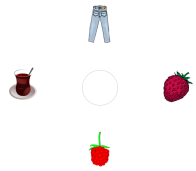

Item Nr.32/3Targets

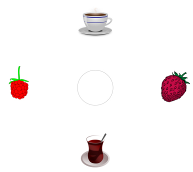

Item Nr.32/4Targets

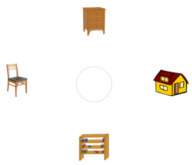

Item Nr.33/0Targets

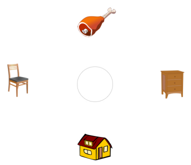

Item Nr.33/1Target

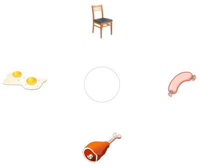

Item Nr.33/3Targets

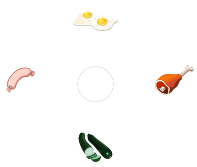

Item Nr.33/4Targets

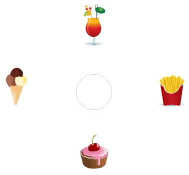

Item Nr.34/0Targets

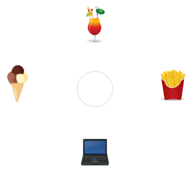

Item Nr.34/1Target

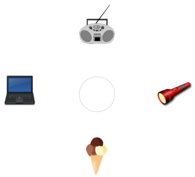

Item Nr.34/3Targets

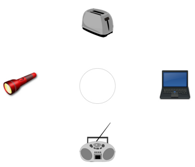

Item Nr.34/4Targets

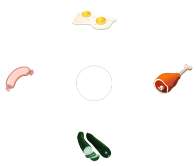

Item Nr.35/0Targets

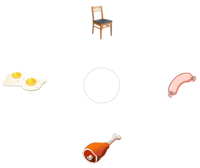

Item Nr.35/1Target

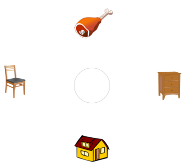

Item Nr.35/3Targets

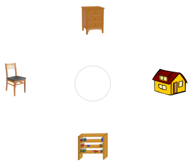

Item Nr.35/4Targets

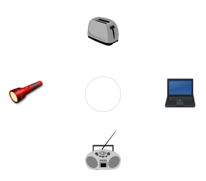
Item Nr.36/0Targets

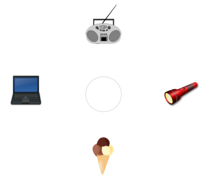
Item Nr.36/1Target

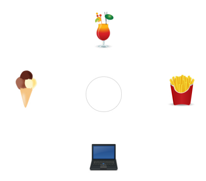
Item Nr.36/3Targets

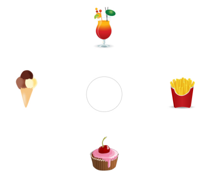
Item Nr.36/4Targets

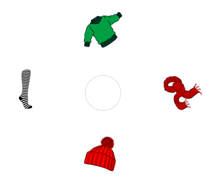
Item Nr.37/0Targets

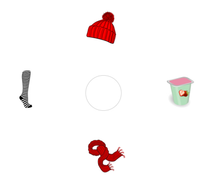
Item Nr.37/1Target

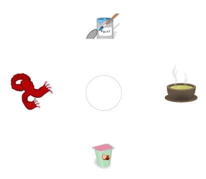
Item Nr.37/3Targets

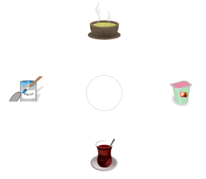
Item Nr.37/4Targets

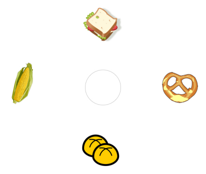
Item Nr.38/0Targets

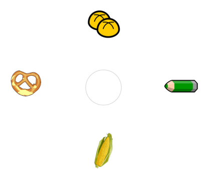
Item Nr.38/1Target

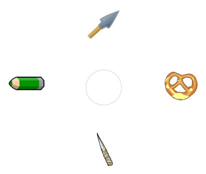
Item Nr.38/3Targets

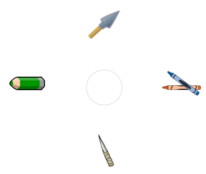
Item Nr.38/4Targets

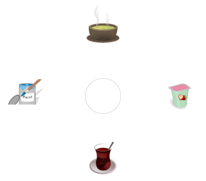
Item Nr.39/0Targets

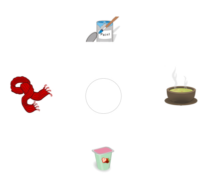
Item Nr.39/1Target

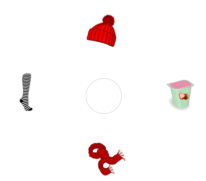
Item Nr.39/3Targets

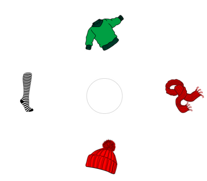
Item Nr.39/4Targets

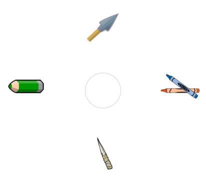
Item Nr.40/0Targets

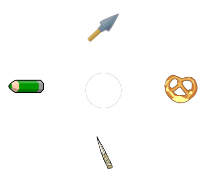
Item Nr.40/1Target

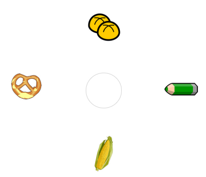
Item Nr.40/3Targets

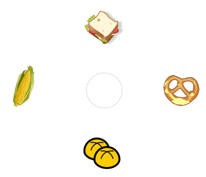
Item Nr.40/4Targets

6 SUPPLEMENTARY TABLES AND FIGURES

Table S4. Sample items and corresponding pretest results for the two nouns in each verb condition: highly constraining (1) and unconstraining (2).

Item	noun	plausibility *M (SD)*	cloze% *M (SD)*
(1) *The man spills soon the*	a) *water*	1.12 (0.68)	13.67 (18.06) [2]
	b) *ice cream*	2.76 (2.17)	0.16 (0.54)
(2) *The man orders soon the*	a) *water*	1.65 (1.50)	< 0.01 (0.0)
	b) *ice cream*	1.90 (1.80)	< 0.01 (0.0)

6.1 Figures

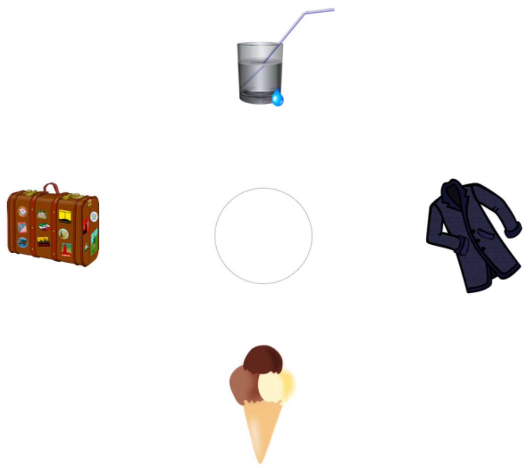

Figure S3: Example of visual stimulus as used in ***Experiments 3 & 4***.

[2] Note that the relevant nouns were pre-selected from the DeReKo corpus (in order to control for frequency) and not collected from the cloze task answers. Hence the relatively high SD for these particular nouns in the cloze task.

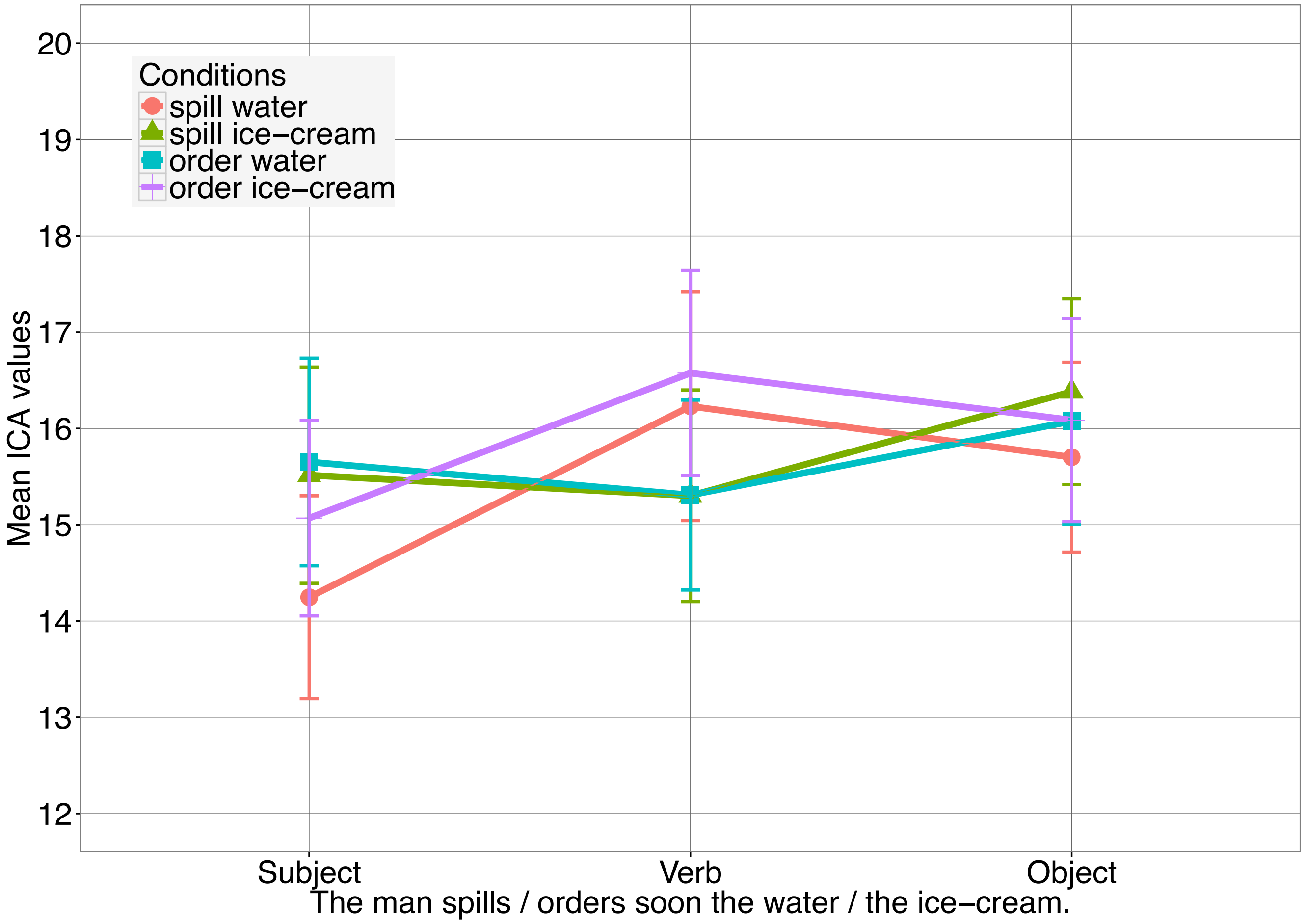

Figure S4: ICA Results for ***Experiment 2*** in all four conditions. Error bars reflect 95% confidence intervals (CI). For the model see 3.

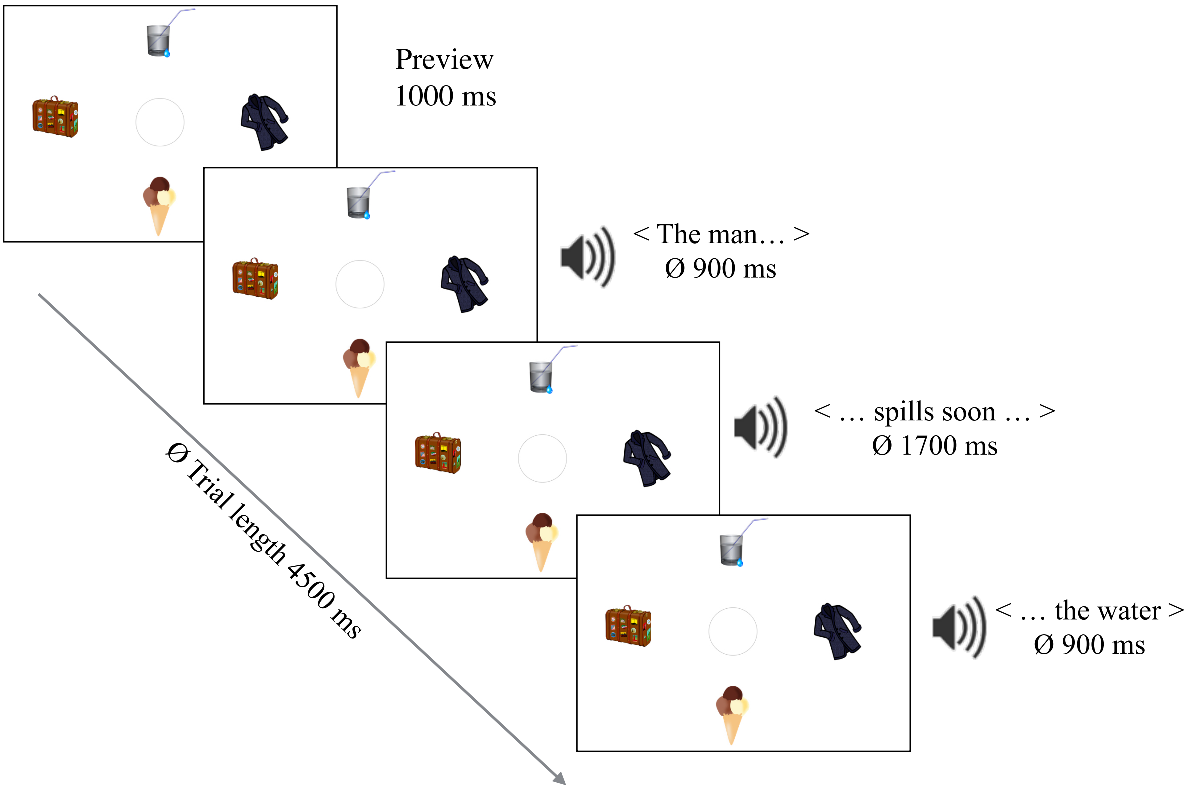

Figure S5: A trial timeline and stimulus example for ***Experiment 3***.

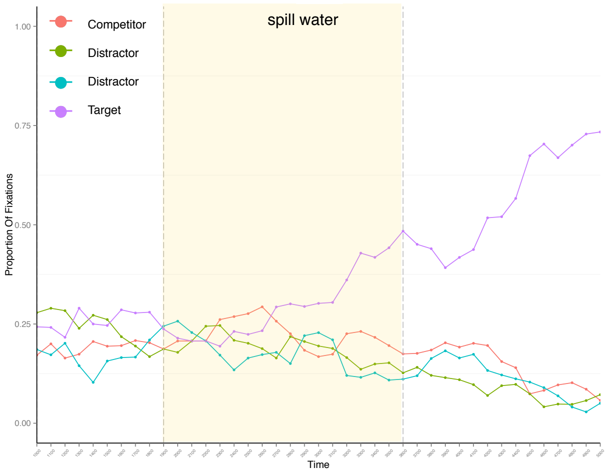

(6) *spill water*

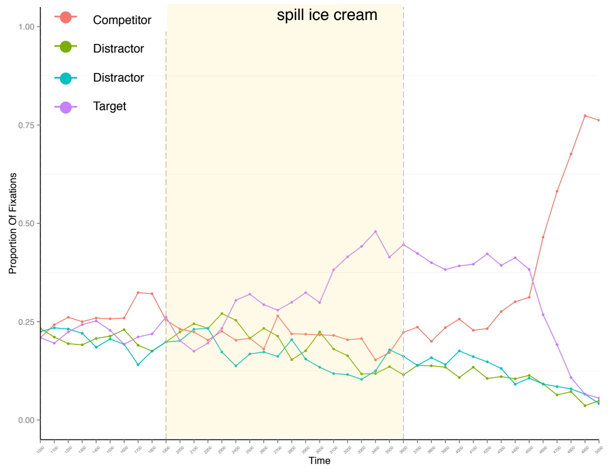

(6) *spill ice cream*

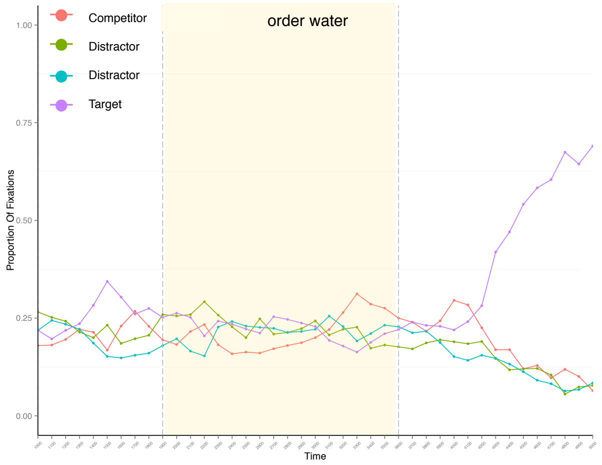

(6) *order water*

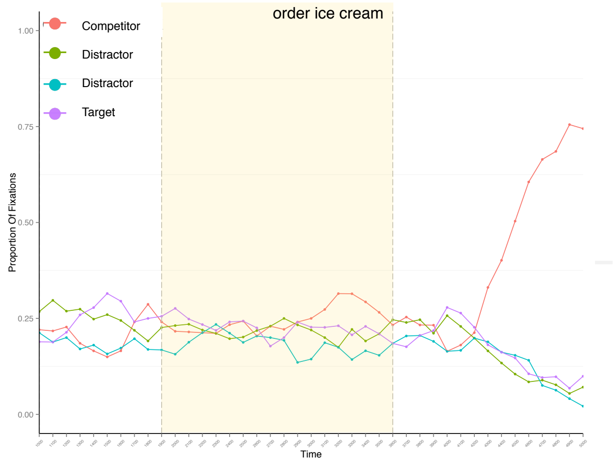

(6) *order ice cream*

Figure S6: Proportion of fixations across trial length in all conditions of ***Experiment 3***

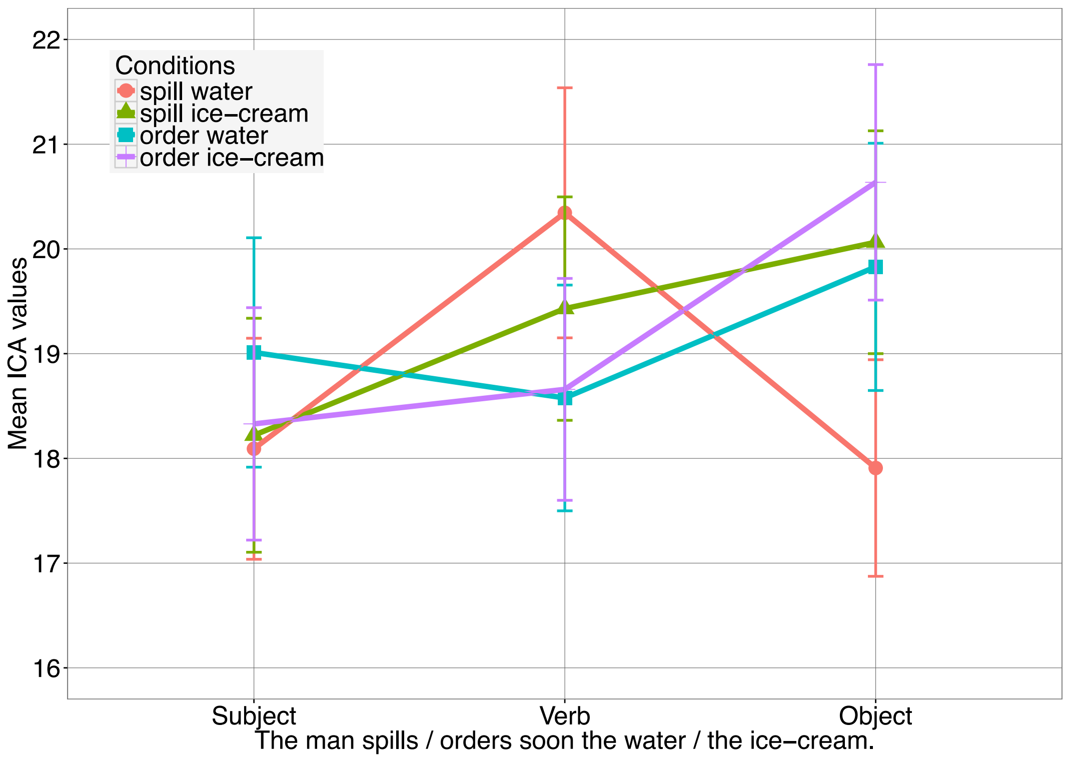

Figure S7: ICA Results for ***Experiment 3*** in all conditions. Error bars reflect 95% confidence intervals (CI). For the models see 3.

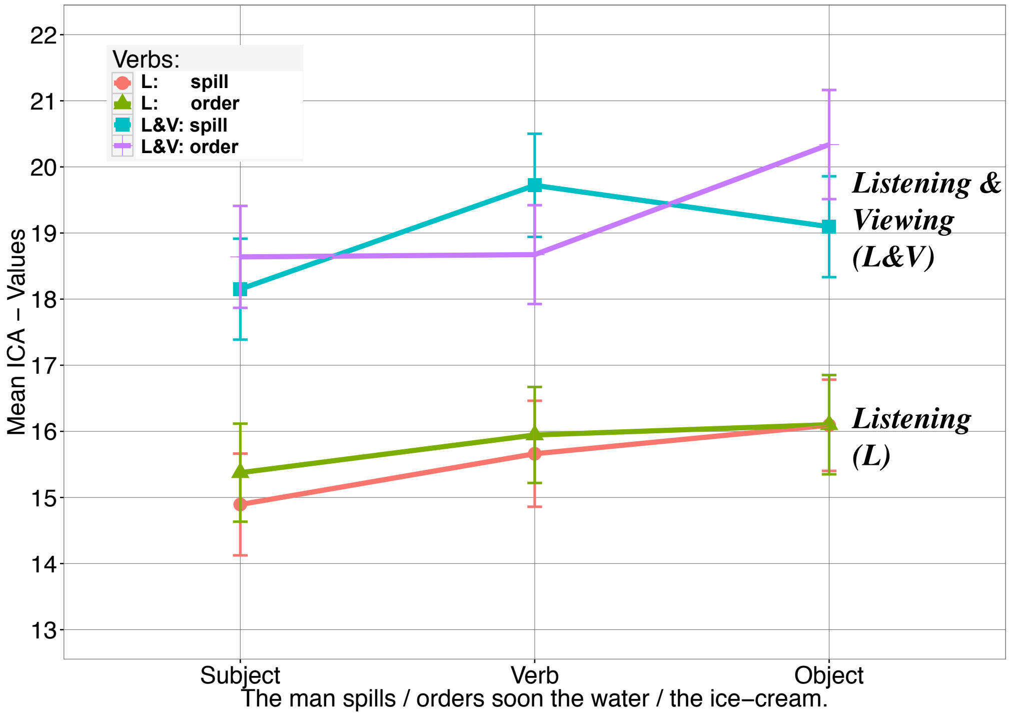

Figure S8: Comparison of ICA values in ***Experiment 2*** & ***Experiment 3***. Both object nouns of a verb condition are considered together for the plot. Graphs show the significant main effect of *Experiment* and a significant interaction of *Verb* and *Experiment*. Error bars reflect 95% confidence intervals (CI). For the models see Section 3.

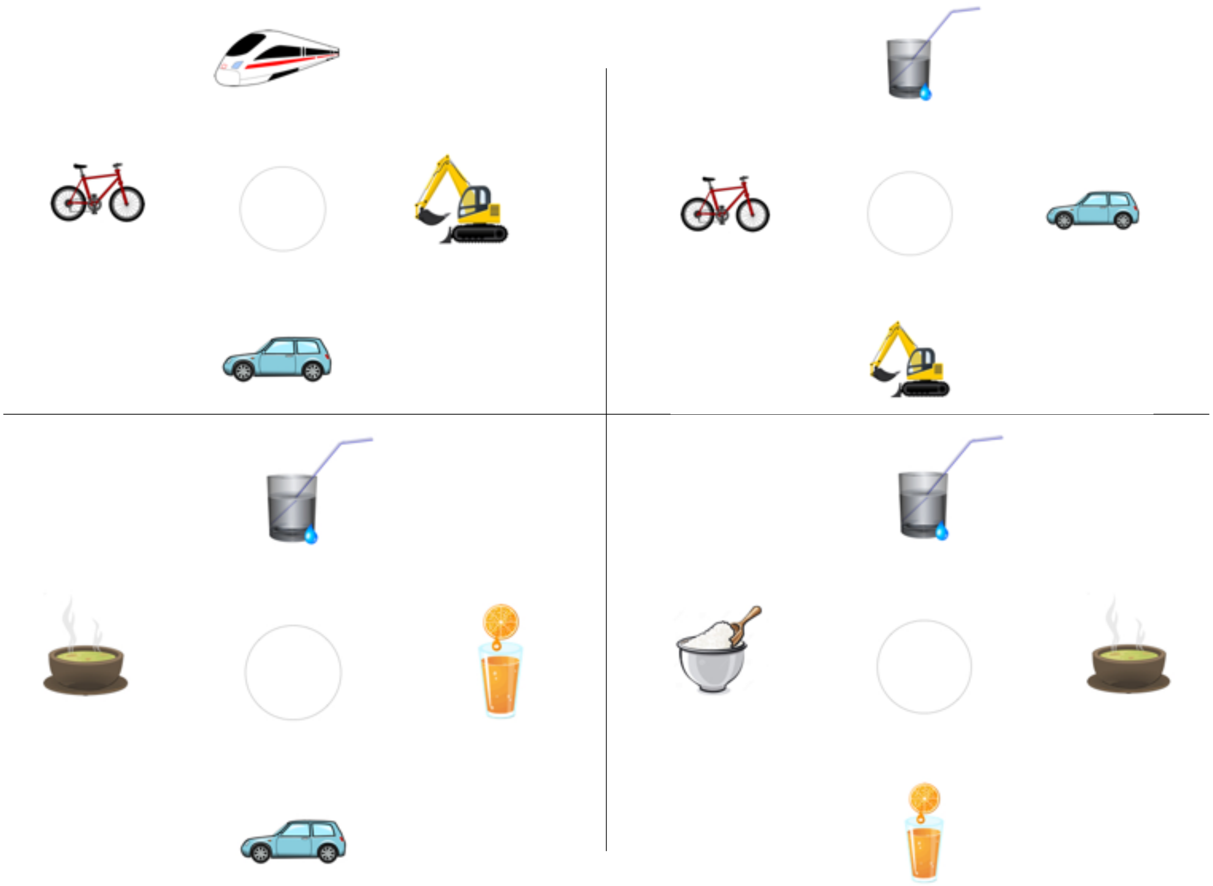

Figure S9: Example stimuli for ***Experiment 4***. From left to right and top to bottom: zero, one, three and four possible targets, given the sentence *“The man spills soon the water.” For all items see 4 and 5.*

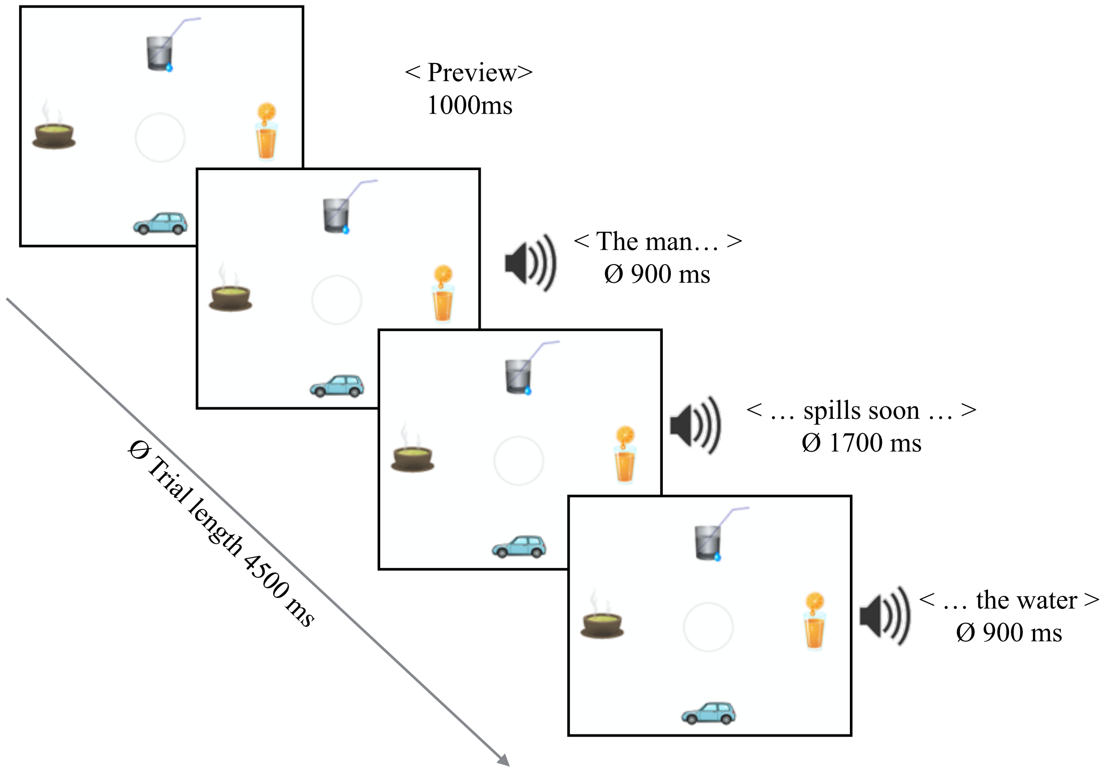

Figure S10: A trial timeline example for ***Experiment 4***. The example scene shows three possible target referents.

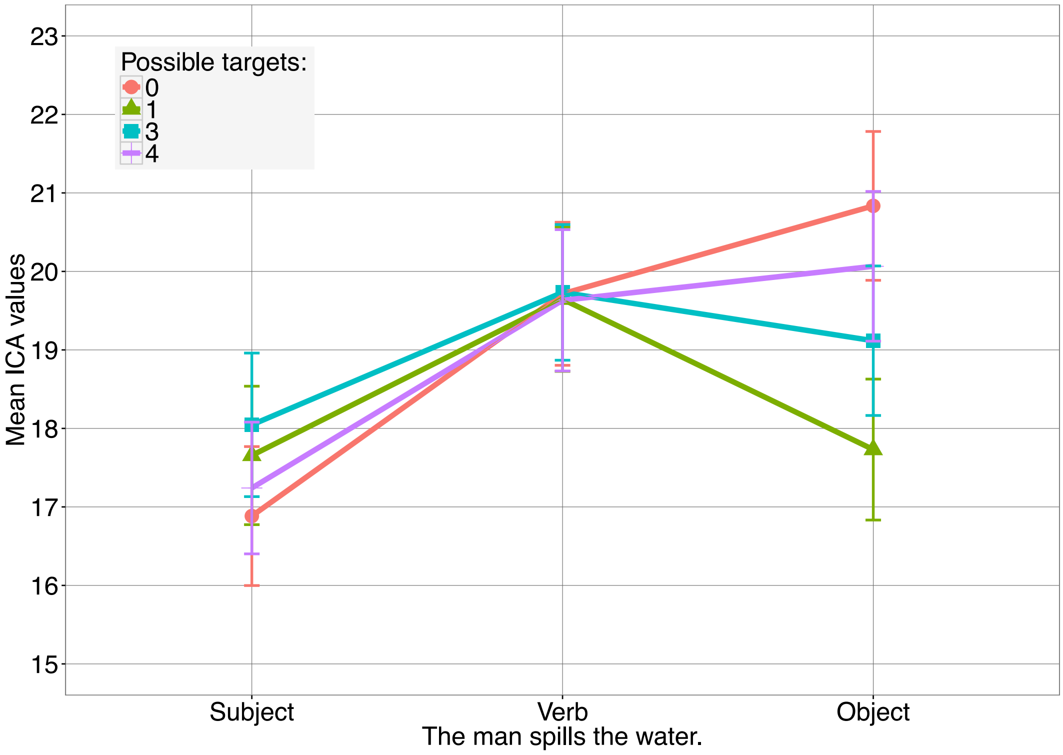

Figure S11: ICA results for ***Experiment 4*** in all conditions. Error bars reflect 95% confidence intervals (CI). For the models see 3.

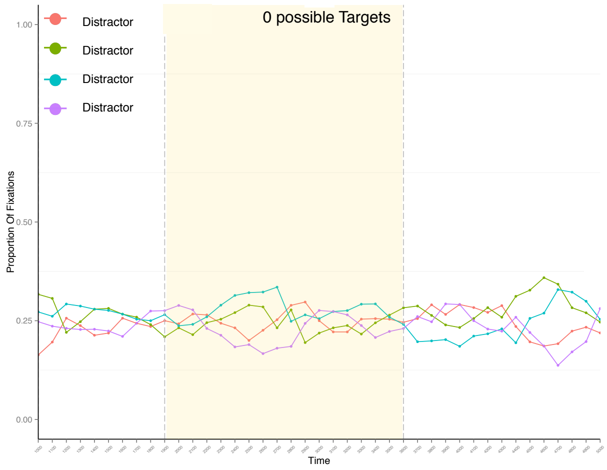

(12) 0 Targets

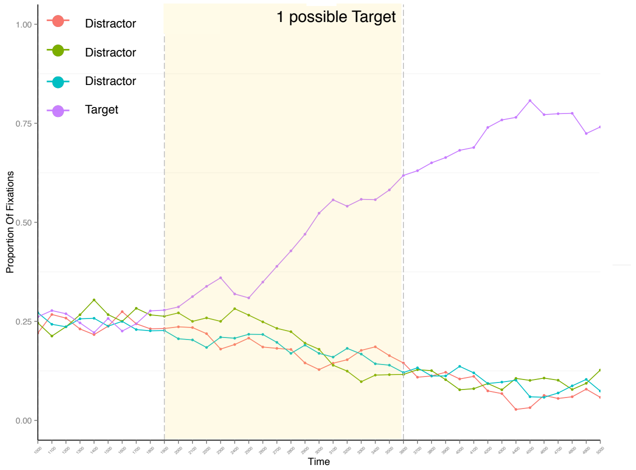

(12) 1 Target

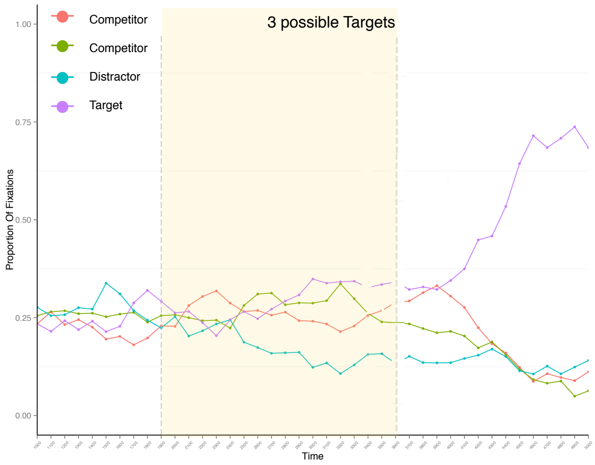

(12) 3 Targets

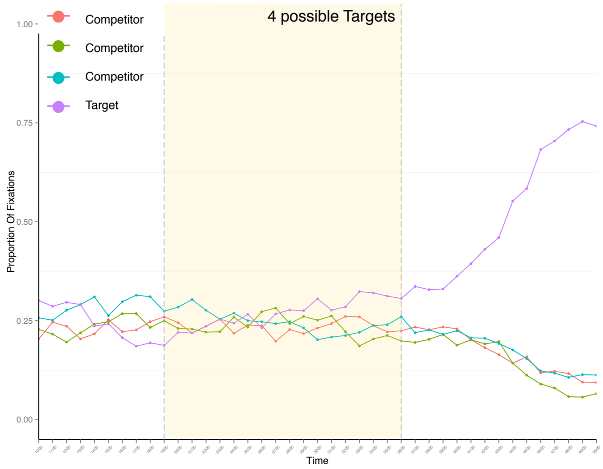

(12) 4 Targets

Figure S12: Proportion of fixations across trial length in all conditions of ***Experiment 4***
